# Treatment of Canine Oral Melanomas: A Critical Review of the Literature

**DOI:** 10.3390/vetsci9050196

**Published:** 2022-04-19

**Authors:** Paolo Pazzi, Gerhard Steenkamp, Anouska J. Rixon

**Affiliations:** Department of Companion Animal Clinical Studies, Faculty of Veterinary Science, University of Pretoria, Pretoria 0110, South Africa; gerhard1.steenkamp@up.ac.za (G.S.); anouska.rixon@up.ac.za (A.J.R.)

**Keywords:** oral, melanoma, dog, surgery, radiotherapy, immunotherapy, chemotherapy

## Abstract

Critical appraisal of the available literature for the treatment of canine oral malignant melanoma (OMM) is lacking. This critical review aimed to evaluate the current literature and provide treatment recommendations and possible suggestions for future canine OMM research. PubMed, Web of Science and Google Scholar were searched in June 2021, for terms relevant to treatment of OMM. Inclusion and exclusion criteria were applied and information on clinical response and outcome extracted. Eighty-one studies were included. The overall level of evidence supporting the various canine OMM treatment options was low. The majority of studies included confounding treatment modalities and lacked randomization, control groups and consistency in reporting clinical response and outcomes. Within these limitations, surgery remains the mainstay of therapy. Adjunctive radiotherapy provided good local control and improved median survival times (MST), chemotherapy did not offer survival benefit beyond that of surgery, while electrochemotherapy may offer a potential alternative to radiotherapy. Immunotherapy holds the most promise in extending MST in the surgical adjunctive setting, in particular the combination of gene therapy and autologous vaccination. Prospective, randomized, double-blinded clinical trials, with a lack of confounding factors and reporting based on established guidelines would allow comparison and recommendations for the treatment of canine OMM.

## 1. Introduction

Melanomas represent one of the most frequently diagnosed tumors in the oral cavities of dogs [1,2,3,4,5]. Oral malignant melanomas (OMM) arise from neoplastic transformation of melanocytes, originating from the neural crest cells and migrating through endodermal and ectodermal mucosa, including the oral mucosa [3,5,6,7,8,9]. Typically, OMM affects older dogs, with the average age at presentation being 11 years [3,5,9,10].

Historically, Cocker Spaniels, Poodles and dogs with heavily pigmented oral mucosa were shown to be at an increased risk for developing OMM [5,11]. However, more recently an overrepresentation of the Chow Chow, Golden Retriever, Labrador Retriever, and Pekingese/Poodle mixed breeds has been reported [2,10]. Although a male gender predisposition has been suggested, many studies demonstrate no gender predisposition [3,5,10,11,12,13].

Oral melanomas are considered to be the most lethal form of canine melanoma, with a reported median survival time of just 65 days in dogs left untreated [9,14,15,16]. A less aggressive form of OMM has been described and questions the true malignant potential of OMM [9]. It has been proposed that the true degree of malignancy of OMM may be less than what their biological behavior suggests, with a study showing only 59% of the 92% of oral melanomas classified to be malignant actually metastasizing or reoccurring [9]. It should also be noted that a different subsection of histologically well-differentiated melanocytic neoplasms (HWDMNs), with a mitotic index of less than three per 10 high-power fields, has been described [17]. Only one study investigated the biological behavior of this particular subset of OMM, reporting a favorable median survival time (MST) of 1020 days post-curative intent surgery [18].

Staging of patients with OMM is performed according to the World Health Organizations (WHO) staging scheme, with stage I patients having a tumor diameter < 2 cm, stage II having a tumor diameter of 2 cm to < 4 cm, stage III having a tumor diameter > 4 cm and/or evidence of lymph node metastasis and stage IV having distant metastasis [19]. Stage of disease, presence of distant metastasis, nuclear atypia, mitotic index, degree of pigmentation, lymphatic invasion and Ki67 index are the parameters that have been shown to have statistical prognostic significance [20,21].

Surgery is currently considered standard of care in the treatment of canine OMM [19]. Traditionally, radiotherapy (RT) and chemotherapy (CT) have been used in the adjunctive setting, with more recent additions to adjunctive therapy including electrochemotherapy (ECT) and immunotherapy [19,22]. To date, no critical review has evaluated the level of evidence for the treatment options of canine OMM. The aim of this critical review was to grade and evaluate the evidence for each treatment option for dogs diagnosed with OMM, and their respective response rates, median disease-free interval (MDFI) or median progression-free interval (MPFI), MST and adverse effects compared to controls or other treatment options. Based on the overall level of evidence for each treatment modality, suggestions for the treatment of OMM will be made and future studies will be proposed.

## 2. Materials and Methods

The sites Pubmed, Web of Science and Google Scholar were systematically searched using the terms ‘melanoma’ AND ‘oral’ or ‘tongue’ AND ‘surgery’ or ‘chemotherapy’ or ‘tyrosine kinase’ or ‘radiotherapy’ or ‘electrochemotherapy’ or’ hyperthermia’ ‘or ‘immunotherapy’ or ‘vaccine’ or ‘gene’ AND ‘dog’ or ‘canine’ to identify relevant references. Only papers published in peer-reviewed journals were considered. Papers published until the end of June 2021 were included.

Studies were excluded if: (1) they were not in English, (2) only an abstract was available, (3) outcomes were not specifically detailed for OMM, (4) results for all types of melanoma were reported as a group and OMM made up less than 80% of the total study population in these studies, (5) clinical outcomes (response rate or MPFI or MDFI or MST) were not detailed, and (6) if the response to therapy of melanoma cell lines was investigated.

Categories of treatment in this review are described based on the primary treatment modality being evaluated in the individual study, with adjunctive therapy reported as appropriate. Where multiple adjunctive therapies were evaluated in a study, the study may be referenced in multiple sections and study details found in the table consistent with the primary treatment modality being investigated. The study type was determined to be prospective or retrospective, randomized or non-randomized, controlled or not controlled, or case series or case report. Studies using historical control groups that were not from the same institution as the treatment group were regarded as case series studies and not clinical trials.

Data extracted from each study included, where available, response rates, MDFI or MPFI, MST and adverse effects. Overall response (OR) was defined as the sum of dogs that showed both a complete response (CR) (complete resolution of measurable tumor) and partial response (PR), a ≥50% reduction in tumor volume with no new tumors. Stable disease (SD) was defined as a <50% change in tumor volume and no new tumors; and progressive disease (PD) was defined as a ≥50% increase in tumor volume or the development of new tumors. Where necessary, times reported in weeks or months were calculated and reported in this review as days (1 month = 30 days). The MST was reported in this review as reported by the individual studies and ranges were included where possible.

Each individual study was assigned a level of evidence (LOE) and each category of treatment was then assigned an overall evidence grade (OEG) according to the previously published format (Table 1) [23], with the following modifications: (1) “non-randomized controlled clinical trial/study” was added to LOE 3b; (2) “case report” was created as LOE 4d; and (3) “non-randomized controlled clinical trial/study” was added to OEG B. Suggestions regarding treatment of OMM in this review are based on the LOE of individual studies and the OEG of treatment groups. Tables are ordered from greatest to least number of animals included in each study. Where peer-reviewed sources were lacking, statements should be considered the opinion of the authors.

## 3. Treatment Review

### 3.1. Surgery Alone

Wide surgical excision has always been the mainstay of treatment for OMM [19,24,25,26]. The OEG for surgical studies was a C (Table 2). Of the 16 studies reviewed, 14 consisted of case series evaluating the effect of surgery alone (CT and/or radiotherapy was inconsistently used as a rescue only in cases where the surgical margins were not clean) [3,16,18,24,26,27,28,29,30,31,32,33,34], and two were control groups for adjunctive therapy (Table 3 and Table 15) [35,36].

Wide resections (unilateral mandibulectomies) compared to partial mandibulectomies did not show decreased local recurrence or metastatic disease [31,37], a finding consistent in other studies [27,30]. Clean surgical margins were associated with an increased MST in one study [24]. However, Tuohy et al. and Hahn et al. did not find a correlation between clean surgical margins and an increased MST [26,38], which may be due to the presence of metastatic disease. Despite clean surgical margins, Sarowitz et al. 2017 reported local tumor regrowth in 11 of the 40 patients [34].

Four studies reported WHO classifications for the enrolled dogs. Of these, the majority of the cases were stage II or III [26,27,30,36]. Only one study reported MST for the dogs according to stage (stage I (*n =* 21)—559 days and stages II and III (*n =* 26)—121 days) and for all stages (*n =* 47)—228 days [36]. One study reported no correlation between tumor stage and survival; however, no data was presented [31].

Median disease-free intervals were recorded in only two studies and they were > 567 days (range not reached) [26] and 152 days (3–2360) [34].

Oral malignant melanoma is a mucosal tumor that often affects the mucosal areas of the oral cavity only, but may also invade the underlying bone [3,16,18,28,33]. Of the studies, five evaluated OMM restricted to the soft tissues only [16,18,29,32,33]. Except for the 1020 day survival in a clearly distinct group of melanomas [18], the MST of the other four studies (222–570 days) are similar to the remaining 11 studies (90–874 days).

Location of the OMM appeared to affect the prognosis for dogs with OMM, despite subtle differences in the individual studies’ definition of the divisions of the oral cavity [3,16,24,25,26,28,34,36]. Hahn et al. 1994 reported a poorer prognosis with caudally situated OMM [38], supported, in part, by Sarowitz et al. reporting surgical excision in the caudal maxillary region had an increased risk for incomplete excisions of any oral tumor [34,38]. In contrast, Schwarz et al. 1991a,b found no significant difference in survival times for OMM, regardless of location, but did show an increased hazard ratio for reduced survival times for any oral tumor situated centrally (OR = 3.4) and caudally (2.3) on the mandible, as well as central (2.6) and caudal (4.3) maxillary tumors [24]. Tuohy et al. found no difference in MST between caudal and rostral locations [26]. In five studies, the OMM was localized to the mandible only and their MST was 180 [28], 219 [30], 240 [24], 270 [27] and 297 (30–1080) days [31]. One study reported on maxillary tumors only with a MST of 225 days [24]. There were three studies reporting only lingual OMM and the MST was 570 [29], 222 [32] and 241 days (4–1037) [33].

Local tumor regrowth and regional and distant metastasis were reported in most of the studies and both entities were commonly found [18,24,25,26,27,28,29,30,31,32,33,34]. Metastasis was common in the regional lymph nodes and the thorax, but spread to other abdominal organs, brain, heart, abdominal wall and appendicular skeleton was also reported [18,24,25,26,27,28,29,30,31,32,33,34].

The complications experienced after wide and radical excisions were inherent to these procedures and were not related directly to the tumor, except where the tumor was still present in the surgical line. The majority of surgical complications were experienced subsequent to caudal maxillary excisions where dehiscence was most commonly reported [24].

The studies evaluated indicated that surgical excision alone provides good MST; however, results are reported without standardized outcomes, making comparison difficult. Very few studies reported the WHO classification of the tumors and the starting point of reported MSTs is not clearly defined. Clean surgical margins and location of the tumors in the oral cavity are two more variables with potentially prognostic value but were seldom reported in a standardized manner. Further studies are required to investigate the potential prognostic value of surgical margin and OMM location.

**Table 2:** Summary of surgical studies evaluating effectivity in oral malignant melanoma.

Overall Evidence Grade: C											
Surgical Protocol	Adjunct	Adjuvant Therapy Treatment Protocol	Study Type	Number of Dogs	WHO Stage of Melanoma	Median DFI (Range) in Days	Median Survival Time (Range) in Days	Local Recurrence and Metastasis	Complications	Reference	LOE
Oral cavity Curative intent surgery	Yes (*n* = 29/70)	CT (7): carboplatin (300 mg/m^2^, IV, q21 d, for 4 to 6 cycles) Metronomic CT (7): combinations of doxycycline (5 to 10 mg/kg, PO, q 24 h), NSAID (piroxicam or carprofen at standard labeled dosages) cyclophosphamide—low dosage (15 to 17 mg/m^2^, PO, q 24 h) Xenogeneic canine melanoma vaccine (1). Combination adjuvant therapy (14): CT, metronomic CT, RT, interferon treatment, and the xenogeneic canine melanoma vaccine	RCS	69/70	I: 36 (51.4%) II: 16 (22.9%) III: 13 (18.6%) IV: 1 (1.4%) Unable to stage: 4	Surgery and adjuvant therapy (29/70): 241 Surgery only (39/70): >567 NR I: (36/70) >567 NR II: (16/70) >187 NR III: (13/70) 245 IV: N/A Location: Rostral (23/70): 360 Caudal (14/70): 358 Margins Complete (51/70): >2310 NR Incomplete (19/70): 446	Surgery and adjuvant therapy (29/70): 396 Surgery only (39/70): 874 I: (36/70) 874 II: (16/70) 818 III: (13/70) 207 IV: N/A Location: Rostral (23/70): 375 Caudal (14/70): 416 Margins Complete (51/70): 619 Incomplete (19/70): 723	LR: 12 M: 25	NE	[26]	4a
Malignant melanomas of lips and oral cavity Surgery alone HWDMNs	No	N/A	RCS	64	Not specified	NE	1020	LR: 2\64	NE	[18]	4a
Soft tissue only Cryo- or conventional surgery 7 dogs, no surgery All sites	No	N/A	RCS	S: 63 NT:7	Not specified	NE	Surgery: 242 No treatment: 65	NE	NE	[16]	4a
Oral melanomas 24 radical excision and 17 conservative (no bone excised)	No	4 cases received cis-diammine- dichloroplatinum II, 2 cases were treated with piroxicam	RCS	41	0: 3 (7%) I: 15 (37%) II: 11 (27%) III: 11 (27%) IV: 1 (2%)	0: 284–765 I: 350–528 II: 0–86 III: 0–56 IV: 706	0: 284–765 I: 415–547 II: 138–179 III: 98–259 IV: 706	NE	NE	[38]	4c
Mandible or maxilla Curative-intent surgery	No	N/A	RCS	40	Not specified	All dogs: 152 (3–2360)	All dogs: 206 (46–435)	LR: 11 M: 12	NE	[34]	4b
Mandible (soft tissue and bone)	No	N/A	RCS	37	Not specified	NE	297 (30–1080)	LR: 8/37 M: 16/37	Drifting, malocclusion	[31]	4b
Lingual tumors Surgery	Yes, 2 cases	Rescue: Carboplatin (*n =* 1) and polyethylated glycol (*n =* 1)	RCS	29	Not specified	NE	241 (4–1037)	LR: 9/29 M: 11/29	For all lingual tumors, not OMM specific: bleeding post-op (10/97), dehiscence (2), partial tongue paralysis (2)	[33]	4a
Surgical treatment All sites	No	N/A	RCS	16	Not specified	NE	90	NE	NE	[3]	4c
Maxilla (soft tissue and bone) Maxillary resections	Yes, 5 cases	RT (10 doses given over 22 days—no Gy given, RT and hyperthermia (42.5 °C for 15 min), non-specific immune modulators, chemo or combinations ONLY as rescue protocol	RCS	14/61	Not specified	NE	225	12 dogs PM: LR: 3 M: 20	Dehiscence (80% caudal)	[24]	4c
Mandible (soft tissue and bone) Mandibulectomies	Yes (46%)	RT (10 doses given over 22 days—no Gy given, RT and hyperthermia (42.5 °C for 15 min), non-specific immune modulators, chemo or combinations ONLY as rescue protocol	RCS	13/81	Not specified	NE	240	9 dogs for PM: LR: 1/9 M: 4 to lymph node, 6 to lungs and 4 elsewhere	Dehiscence, prehension dysfunction, medial drift, ptyalism	[25]	4c
Lingual tumors Surgery alone	No	N/A	RCS	11/42	Not specified	NE	222 (47–840)	LR: 2/11 M: 5/11	Ptyalism Dehiscence	[32]	4c
Mandible (bone and soft tissue) Mandibulectomies	No	N/A	RCS	10	I: 2 (2%) II: 6 (6%) III: 2 (2%)	NE	180	LR: 1/10 M: 3/10	NE	[28]	4c
Lingual	Yes, 2 cases	1 case received 36Gy RT and hyperthermia 1 case received Dimethyl-trianzeno-imadozole- carboxamide (200 mg/m^2^ BSA for 5 days, plus BCG (unknown dose) prednisone (20 mg/m^2^ BSA) sid	RCS	7/57	I: 1 (14%) II: 4 (57%) III: 2 (29%)	NE	570	M: 3/7 LR: 3/7	NE	[29]	4c
Mandible (bone and soft tissue) Partial mandibulectomy Controls local recurrence	Yes, 1 case	1 case received RT and hyperthermia	RCS	7/30	II: 1 (14%) III: 6 (86%)	NE	219	LR: 1/7 M: 5/7	NE	[30]	4c
Mandibular, mandibulectomies	Yes, 3 cases	3 cases received *C. parvum*	RCS	4	III: 4	NE	270	M:3/4	Ptyalism, cheilitis	[27]	4c

Abbreviations: CT: chemotherapy, DFI: disease-free interval, HWDMNs: histologically well-differentiated melanocytic neoplasms, LR: local recurrence, M: metastasis, N/A: not applicable, NE: not evaluated, NR: not reached, NT: no treatment, PM: postmortem, RCS: retrospective case series, RT: radiotherapy, S: surgery

### 3.2. Chemotherapy

Chemotherapy is usually considered as any drug or chemical substance used to treat cancer that is toxic to neoplastic or rapidly dividing cells, with tyrosine kinase inhibitors included—despite them not being cytotoxic [39]. The ideal chemotherapeutic agent would have selective toxicity, high distribution to tumor burden, be devoid of tumor resistance development and be non-toxic to the patient [40].

With the exception of two studies [35,37], the studies in which CT were used as a sole agent were all case series or case reports without the inclusion of a control treatment group [26,41,42,43,44,45,46,47,48,49]. The OEG of CT studies for OMM was a C (Table 3). Most studies (8/12) consisted of twenty dogs or less. When described, the WHO classification showed that the majority of dogs in these studies were in stage II or III. Drugs evaluated included carboplatin (30, 25, 17 and 1 dog) [35,41,43,48], intralesional cisplatin implants (20) [42], masitinib mesylate (14) [47], mitoxantrone (12) [44], cisplatin in combination with piroxicam (11) [45], artesunate (3) [46], olomoucine (1) [49]; in one study, individual dogs received one of the following: carboplatin (21), platinum-based treatment (5), lomustine (1), dacarbizine (1), doxorubicin (1) and metronomic chemotherapy (4) [37]; in another study, individual dogs received one of the following: carboplatin (7), metronomic CT (7), xenogeneic canine melanoma vaccine (1), combination therapy (14) of CT, metronomic CT, RT, interferon treatment, and the xenogeneic canine melanoma vaccine [26].

Response rate was reported in 6/12 studies, and overall response was highest in dogs treated with intralesional cisplatin (60%) [42]. Despite the good OR, MST was only 116 days in that study. All other studies had a poor response rate of less than 20%. Boria et al. reported that after multivariate analysis, only cisplatin dose (mg/kg) was significantly associated with response [45]. Mitoxantrone did not appear to be an effective chemotherapeutic, with only 1/12 cases going into partial remission [44].

Median survival times should be evaluated based on whether the study considered survival time from the point of diagnosis, surgery, or at the point of the institution of adjunctive therapy. For studies in which MST was reported (8/12), carboplatin afforded the longest survival times (440 and 389 day, respectively) [35,43], but these were defined as survival from the point of diagnosis. Boston et al. reported a MST of 353 days from the point of surgery [37]. Mastinib, cisplatin in combination with piroxicam and intralesional cisplatin resulted in what appear to be poorer MSTs (119, 119 and 116, respectively) [42,45,47], but the survival in these studies were taken from the initiation of CT, most commonly in dogs with non-resectable tumors or after recurrent disease. The majority of the dogs (26/45) in those three studies received surgery (or another intervention) before CT and the time from surgery to CT was not included in the MST. The lack of standardized reporting and specific outcomes of dogs in the studies that combined dogs with and without prior surgery, makes evaluation of the effect of prior surgery and subsequent CT compared to only CT impossible. The exception to this is Brockley et al. and Boston et al. who identified no significant differences in dogs that underwent only surgery (495 and 335 days, respectively) compared to dogs that underwent surgery and carboplatin therapy (389 and 352 days, respectively) [35,37]. Interestingly, Brockley et al. showed that carboplatin makes no significant difference to survival if gross (macroscopic) disease is present (184 days) compared to palliative therapy alone (141 days). Dank et al. found that stage of disease, treatment with RT therapy and dosage of carboplatin were not associated with a shorter progression-free survival or overall survival [43]. The lack of OR and low MST in treated dogs may be related to dose reductions and subsequent lowered median dose delivered due to varied chemotherapeutic toxicities in three of the studies [35,41,43]. Tuohy et al. found that dogs receiving adjuvant therapy (14/29 receiving a form of adjunctive CT) after surgical excision had a higher hazard of disease progression but not death, compared with dogs that did not receive adjuvant therapy after adjustment for tumor size and presence of metastases at diagnosis [26]. Mastinib was evaluated in OMM cases with advanced disease (stage III and IV only) that were progressive despite conventional treatment with surgery or RT, despite the advanced disease, survival was comparable to the combination of cisplatin and piroxicam as well as intralesional cisplatin implant, of which the majority were stage II and III [42,45,47].

The majority of adverse events for systemic CT were considered mild to moderate, and almost all were self-resolving. Carboplatin was associated with the highest grade of complication, all of which involved gastrointestinal toxicosis—vomiting or diarrhea (for which two dogs were euthanized), or hematological toxicities such as neutropenia and thrombocytopenia [35,41,43]. Local intralesional cisplatin was associated with local necrosis limited to the implant site or oral ulceration, three dogs developed oronasal fistulas and another two trismus that resolved after a few weeks [42].

From the limited studies available, that lacked uniformity in design, control groups, and reporting of response and survival variables, it would appear that the inclusion of chemotherapeutics after surgery for non-resectable or progressive tumors does not offer significant survival benefit beyond that of surgery [26,35,37,43]. Further studies with Mastinib, Toceranib and other chemotherapeutics are required.

### 3.3. Radiotherapy with Adjunctive Therapy

Radiotherapy utilizes ionizing radiation to control or kill cancer cells and is normally delivered by a linear accelerator. It has long been utilized for adjunctive therapy of sarcomas and carcinomas in veterinary medicine.

Two of the studies in which RT was used with CT as an adjunctive included a control group that received only RT to evaluate the effect of CT in combination with RT, although the RT-only groups were small (Table 4) [50,51]. Three studies, were retrospective case series [52,53,54]. One study used the melanoma vaccine as an adjunctive in 9/11 (82%) of dogs [55]. The OEG of the RT with adjunctive therapy studies for OMM was a C. Two studies consisted of over 100 dogs [53,54], while the remainder had less than 40 dogs per study, all at various WHO stages, and the majority of dogs classified at stage II and III.

Treatment regimens varied from 3 fractions to up to 8 fractions. Total Gys varying from 24 to 50 Gy, depending on study design and intention with therapy. Carboplatin was the most commonly used systemic chemotherapeutic, followed by cisplatin and only one study used melphalan [50,51,52,54]. Cisplatin was used as a local chemotherapeutic in one study [53].

Radiotherapy is more effective on microscopic disease than macroscopic disease [56]. Only one study did not include surgery before the initiation of RT, meaning only gross disease was present in that study [50]. The remaining studies varied widely with the number of dogs that received surgery before RT and the number of dogs treated that had only microscopic disease. In studies where more than one chemotherapeutic was administered to different dogs, the survival was reported as a group rather than per treatment, making identification of individual treatment advantages impossible [26,52,53,54]. Kawabe et al. compared orthovoltage x-ray (OVX), megavoltage x-ray (MVX) and electron beam RT, but the results were difficult to interpret as 52/111 dogs received local (26) or systemic therapy (26) in addition to the RT [53].

**Table 3:** Summary of chemotherapeutic studies evaluating effectivity in oral malignant melanoma.

Overall Evidence Grade: C												
Chemotherapeutic Evaluated	Initial Surgery or Other Treatment	Chemotherapy Treatment Protocol	Adjunctive Therapy	Study Type	Number of Dogs	WHO Stage of Melanoma	Response Rate	Median PFI (Range) in Days	Median Survival Time (Range) in Days	Adverse Events	Reference	LOE
Carboplatin	LRC: 17 Surgery: 11 Surgery and carboplatin: 6 GD: 13 Carboplatin only: 8 Palliative: 5	Planned: 300 mg/m^2^ q21d, 4–6 treatments Actual: mean dose: 288 mg/m^2^, median treatments: 4 (range 1–11) Dose reduced by: 20%—2 dogs 10%—2 dogs	No	RCCS	30 LRC: 17 GD: 13	I: 9 (30%) II: 11(37%) III: 9 (30%) IV: 1 (3%)	NE	NE	All OMM: 389 (251–527), from diagnosis I: 242 (292–556) II: 246 (56–436) III: 495 (363–627) IV: 147 LRC Surgery: 495 (246–1460) Surgery and carboplatin: 389 (21–560) GD Carboplatin: 184 (93–275) Palliative: 141 (6–276)	Neutropenia Grd 4: 2 Death (severe gastritis and azotemia): 1	[35]	3b
Carboplatin	Initially surgical resection: 13 Radiation prior to carboplatin: 7	Planned: 300 (16 dogs) -350 (11 dogs) mg/m^2^ IV until no further response observed. Actual: median treatments: 2 (range 1–18) Dose reduced by 25% in 2 dogs (from 300 mg/m^2^)	No	RCS	25	I: 2 (8%) II: 3 (12%) III: 13 (52%) IV: 7 (28%)	OR: 7/25 (28%) CR: 1 (4%) PR: 6 (24%) SD: 9 (36%) PD: 9 (36%)	66	NE	300 mg/m^2^: Anorexia Grd 1: 1 Grd 2: 3 GI toxicity Grd 2: 2 Grd 3: 3 Grd 4: 3 350 mg/m^2^: Vomiting Grd 2: 1	[41]	4b
Intralesional cisplatin implants	Surgical debulking: 12 Cryosurgery: 1 Dacarbazine: 1	Weekly implants: mean 5.2 treatments (range 2–15), 17 cisplatin only Mean dose of cisplatin/treatment: 4.9 mg/cm^3^ (range 0.3–22.1)	1 MTX after cisplatin implants; 2 MTX followed by carmustine	PCS Only measurable tumors were included	20	I: 4 (20%) II and III: 15 (75%) IV: 1 (5%)	OR: 12/20 (60%) CR: 9/20 (45%) PR: 3/20 (15%) SD: 2 (10%) PD: 6 (30%)	NE	116	Necrosis limited to implant site: 17, Ulceration: 14 Oro- nasal fistula: 3 Fibrosis of the jaw with trismus with resolution: 2	[42]	4b
Carboplatin	Surgery: 17	Actual: Median of 300 mg/m^2^ (range 150–300), median of 4 (range 2–11) treatments 300 mg/m^2^: 11 dogs 250–300 mg/m^2^: 4 dogs 150–250 mg/m^2^: 2 dogs	RT: 11 8 Gy q7d, 4 fractions: 5 dogs 6 Gy q3–4d, 6 fractions: 4 dogs 10 Gy q7d, 3 fractions: 1 dog 6 Gy q7d, 4 fractions: 1 dog	RCS	17	I: 2 (12%) II: 9 (53%) III: 4 (23%) Not staged: 2 (12%)	NE	All: 259 (CI: 119–399) Sx and CT: 210 Sx, RT, CT: 291	All: 440 (CI: 247–633), from diagnosis	Neutropenia Grd 1: 1 Grd 2: 1 Grd 3: 1 Grd 4: 3 Thrombocytopenia Grd 1: 1 Renal toxicity Grd 1: 1 GI toxicity Grd 1: 2 Grd 2: 2	[43]	4c
Mitoxantrone	Yes, numbers for OMM not specified	Initially: 2.5 mg/m^2^, increased to 4–5 mg/m^2^ in 0.5 mg/m^2^ increments. Total of 1–5 doses	Yes, numbers for OMM not specified	PCS All dogs had measurable tumors	12	Not specified	OR: 1 (8%) CR: 0 PR: 1 (8%) SD or PD: 11 (92%)	Remission time for PR: 21 days	NE	NE	[44]	4c
Cisplatin and piroxicam	No Only dogs with non- resectable tumors included	Piroxicam (0.3 mg/kg, PO, q24 h) from 5 days before cisplatin. MTD of cisplatin with piroxicam was 50 mg/m^2^ IV every 3 weeks with standard saline diuresis	No	PCS (Phase I & II clincal trial—pharmaco-kinetic study)	11	Not specified	OR: 2/11 (18%) CR: 2/11 (18%) PR: 0 SD: 1/11 (9%) PD: 8/11 (73%)	NE	119 (10 to 370)	Not specifically evaluated for OMM. Renal toxicity in 7/20 dogs in study	[45]	4c
Artesunate	No Only dogs with non- resectable tumors included	First five days increased stepwise from 600 to 1000 mg/m^2^/day, maintained till day 7–14. If no adverse effects, increased to 1200 mg/m^2^. 3 OMM cases: 688, 895 and 938 mg/m^2^/kg	No	PCS (Safety/efficacy field study)	3	Not specified	SD: 1 (day 25) SD: 1 (day 14, PD at day 42) Treatment stopped at 10 days: 1 (response unknown)	NE	NE	Fever Grd 3: 1 GI toxicity Grd 1: 1 Grd 2: 1	[46]	4c
Carboplatin	Surgical resection	250 mg/m^2^, IV, q3 week	No	Case report	1	IV	NE	NE	90	None	[48]	4d
Cell cycle inhibitors												
Masitinib mesylate	Various combination of surgery or radiotherapy Enrolled due to progressive disease	Standard dose: 12.5 mg/kg (mean dose 12.08 mg/kg) PO, q24 h	Xenogeneic human tyrosinase DNA canine melanoma vaccine (Oncept^®^): 6	PCS	14 OMM, 2 digital, 1 anal	III: 4 (29%) IV: 10 (71%)	OR: 2/14 (14%) PR: 2 (14%) SD: 6 (43%) PD: 6 (43%)	All dogs: 66 (25–124)	All dogs: 119 (21–255)	Anemia Grd 2: 1 Neutropenia Grd 1: 1 Anorexia Grd 2: 1 Diarrhea Grd 1: 1	[47]	4c
Olomoucine (cyclin- dependent kinases)	No	Olomucine at 8 mg/kg/day IV, q24 h, for 7 days	Yes, debulking surgery to remove necrotic tumor	Case report	1	III: 1	CR	NE	Dog died 3 weeks after initiating therapy, post-operatively	Severe necrosis of mass	[49]	4d

Abbreviations: CI: confidence interval, CR: complete response, CT: chemotherapy, GD: gross disease, GI: gastrointestinal, Grd: grade, Gy: Gray, LRC: loco-regional control, MTD: maximum tolerable dose, MTX: methotrexate, NE: not evaluated, OR: overall response, PCS: prospective case series, PD: progressive disease, PFI: progression-free interval, PO: per os, PR: partial response, RCCS: retrospective controlled clinical study, RCS: retrospective case series, and SD: stable disease.

Similar OR response was reported across the studies and ranged from 73% to 93%. Dogs that underwent RT only had similar overall response (77%) compared to dogs that received adjunctive carboplatin (81%) [50]. At the four–eight-week post-RT re-examination time point, the proportion of dogs showing either a complete or a partial response to therapy was not significantly different between the RT alone and adjunctive carboplatin [50]. Prolux et al. reported that neither the administration of CT nor the RT therapy protocol used had a significant effect on local response [54], and systemic CT was not related to the development of metastasis in univariate analysis. When comparing the MPFI with treatment response with the type of treatment, no statistically significant differences were observed [51].

Median survival time for RT and an adjunctive chemotherapeutic in the reported studies was between 134 [55] and 396 [26] days. Proulx et al. reported that the administration of systemic CT (carboplatin or melphalan) had no effect on the time to first event, the development of pulmonary metastasis, or survival [54]. Additionally, Murphy et al. showed no evidence of beneficial effect of carboplatin therapy in conjunction with RT on median survival (MST: 286 days) over RT alone (MST: 307) [50]. The median dose of chemotherapeutic, particularly carboplatin, was below the recommended dose in three of the larger studies and may be responsible for the lack of response, and higher doses may result in longer MSTs [50,53,54]. The MST of dogs receiving only RT in these studies is similar to dogs in other studies receiving only RT, although radiotherapy protocols differed [57,58]. Tuohy et al. showed 14/29 dogs receiving a combination of adjunctive RT, CT and/or immunotherapy after surgical excision had an earlier likelihood of disease progression, but not death, compared to dogs that did not receive adjuvant therapy after adjustment for tumor size and presence of metastases at diagnosis [26]. In contrast, Cunha et al. showed dogs receiving surgery/CT/RT had a greater MST (380 days), followed by dogs treated with CT/RT therapy (150 days), and finally, than those treated with RT alone (60 days) [51]. Unfortunately, there were only three dogs in the RT-only group and all were in stage IV while the 4/5 dogs receiving surgery/CT/RT were in stage III and one was in the stage II. Additionally, dogs at stage II had a significantly longer survival time when compared to dogs at stage IV [51]. When evaluating the 107 dogs in one study that received OVX (68 dogs) or MVX (39 dogs) therapy, a significantly longer survival was identified with MVX (233 days) compared to OVX therapy (121.5 days) [53]. In the same study, when WHO classification was evaluated, only dogs with stage III OMM showed significance difference between survival if OVX or MVX was used, with MVX showing longer survival. As would be expected, MST differed significantly between dogs with stage I disease and those with all other disease stages [53].

Grade 1 and 2 acute RT side effects were most commonly reported, with only one study reporting hematological and gastrointestinal toxicities from chemotherapeutics (specifically carboplatin) [50,51,52,53]. Adverse side effects do not appear to be a reason to withhold RT and adjunctive chemotherapeutics.

Due to the heterogeneity of study design and dosages of RT and chemotherapeutics, clear treatment guidelines cannot be provided. Based on the available evidence, although overall response to RT with an adjunctive chemotherapeutic was good, MST varied widely. Two studies identified no advantage of chemotherapeutics over RT alone while the only study that did find a benefit over RT alone, consisted of only three dogs in the RT-only group. The available evidence does not support the use of adjunctive chemotherapeutics over RT alone to improve MST, but further studies are required with chemotherapeutics administered at higher dosages. If a patient suffers from Stage III OMM, MVX RT should be prioritized.

**Table 4:** Summary of radiotherapy with adjunctive therapy studies evaluating effectivity in oral malignant melanoma.

Overall Evidence Grade: C											
Radiotherapy Treatment Protocol	Initial Surgery	Chemotherapy Treatment Protocol	Study Type	Number of Dogs	WHO Stage of Melanoma	Response Rate	Median PFI (Range) in Days	Median Survival Time (Range) in Days	Adverse Events	Reference	LOE
(1) 30 Gy in 69 (49%) dogs at 3 × 10 Gy fractions on day 0, 7 and 21 (2) 36 Gy in 54 (39%) dogs at 4 × 9 Gy fractions on day 0, 7, 14 and 21 (3) Median of 46 Gy in 17 (12%) dogs with varied fractionation schemes	Yes: 84 At RT initiation: 93 (66%) had macroscopic tumor; 47 (34%) had microscopic tumor	CT: 80 (57%) - Carbo (225 mg/m^2^, IV, q 3 weeks): 60 dogs - Melphalan (0.5 mg/kg, IV, q4 weeks): 17 dogs - Cisplatin (40 mg/m^2^, IV): 3 dogs	RCS	140/150	<III: 62 (42%) III: 69 (49%) IV: 9 (6.4%)	86 dogs OR: 71 (82%) CR: 44 (51%) PR: 27 (31%) SD: 14 (16%) PD: 1 (1%)	All dogs: 150	All dogs: 210	NE	[54]	4a
OVX (68): 40–50 Gy at 6.3–10.0 Gy/fraction in 4–6 fractions at 7–10 day intervals MVX (39): 40–50 Gy at 6.0–10.0 Gy/fraction in 4–8 fractions at 7–10 day intervals EBR (4): 6.0 Gy/fraction in 6 fractions at 7 day intervals, total dose of 36 Gy	Yes Surgery: 45 Surgery and CT: 27/111	Cisplatin (0.5 mg/dog/treatment, q 1 to 2 wk) injected directly into the tumor Carboplatin (180–250 mg/m^2^, IV, q 3 weeks) Local treatment only: 26 Systemic treatment only: 26 Both: 14	RCS	111	I: 19 (17%) II: 24 (22%) III: 37 (33%) IV: 31 (28%)	87 dogs OR: 74 (85%) CR: 38 (44%) PR: 36 (41%) SD: 7 (8%) PD: 6 (7%)	NE	All dogs: 171 (3–1620) I: 758 II: 278 III: 163 IV: 80 OVX: 121.5 (11–1620) MVX: 233 (3 -966)	Acute radiation damage:49 OVX: Toxicity scores Grd: 1 = 18 Grd 2 = 14 Grd 3 = 4 MVX: Toxicity scores Grd 1 = 31 Grd 2 = 21 Grd 3 = 5 EBR Toxicity scores Grd 1 = 1 Grd 2 = 1	[53]	4a
36 Gy in 6 weekly 6-gray (Gy) fractions of megavoltage irradiation Administered 60 min after administration of platinum-containing CT	Yes Incompletely resected OMM with no identifiable metastasis included	Cisplatin (36 dogs): 10–30 mg/m^2^ IV during 4 h saline and mannitol diuresis Carboplatin (3 dogs): 90 mg/m^2^, IV, over 30–45 min	RCS	39	I: 22 (56%) II: 3 (8%) III: 14 (36%)	NE	139 (20–1077)	All dogs: 363 (24–2163)	Grd 1 acute radiation effects Grd 1 chronic radiation effects	[52]	4b
36 Gy in four weekly fractions of 9 Gy (to primary and metastatic nodules)	No	Planned: 2–6 doses carboplatin at 300 mg/m^2^ q 21 days Actual: median: 282 mg/m^2^, median of 3 doses (range 2–6). All doses at 300 mg/m^2^: 6/15	RCCS	28 RT only: 13 RT and CT: 15	RT only: I: 2 (15%) II: 7 (54%) III: 3 (23%) Unknown: 1 (8%) RT and CT: I: 1 (7%) II: 6 (40%) III: 7 (46%) Unknown: 1 (7%)	RT: OR: 10 (77%) CR: 7 (54%) PR: 3 (23%) SD: 1 (8%) PD: 2 (15%) RT and CT: OR: 13 (81%) CR: 8 (50%) PR: 5 (31%) SD: 1 (6%) PD: 2 (13%)	NE	RT: 307 (108–585) RT and CT: 286 (87–707)	Neutropenia: Grd 1: 5 Grd 2: 1 GI toxicity: Grd 3: 1 RT Mucositis Grd 1: 4	[50]	3b
(1) 24 Gy in three weekly sessions of 8 Gy in 16 cases (2) 32 Gy in four weekly sessions of 8 Gy in 8 cases	Yes Prior surgery: 6 As part of therapy: 5	Carboplatin (250–300 mg/m^2^), IV, q21–30 days, total of 4 doses. First dose given 5–7 days before start of RT	RCCS	24 RT and CT: 15 Surgery then RT/CT: 3 RT only: 3 ECT and RT: 1	I: 1 (4%) II: 4 (17%) III: 12 (50%) IV: 7 (29%)	OR: 14 (93%) CR: 4 (26%) PR: 10 (67%) SD: 1 (7%)	CR: 213	RT: 60 RT and Carbo: 150 Surgery, CT and RT: 380 I: 390 II: 277 III: 120 IV: 90	Numbers not specified: Cutaneous: Grd 1	[51]	3b
(1) 8-Gy fractions q7 days for 4 weeks (2) 6-Gy fractions q 3 or 7 days for 6 weeks (3) 3.5-Gy fractions q 3 days for 2 consecutive days	Yes: 7 Only gross disease included	Melanoma vaccine as adjuvant treatment in 9/11	RCS	11	Not specified	SD: 8 (73%) PD: 3 (27%)	NE	134 (21–451)	Not specified for OMM	[55]	4c

Abbreviations: CR: complete response, CT: chemotherapy, ECT: electrochemotherapy, EBR: electron beam radiotherapy, Grd: grade, Gy: Gray, MVX: megavoltage x-ray, NE: not evaluated, OVX: orthovoltage x-ray, OR: overall response, PCS: prospective case series, PD: progressive disease, PFI: progression-free interval, PR: partial response, PRT: palliative radiation therapy, RCCS: retrospective controlled clinical study, RCS: retrospective case series, RT: radiotherapy, and SD: stable disease.

### 3.4. Radiotherapy without Adjunctive Therapy

Three prospective, and one retrospective case series were included in the evaluation of RT without adjunctive CT (Table 5) [57,58,59]. One study included dogs that did not have macroscopic disease recurrence after initial surgery and the study design was to identify prognostic factors in dogs treated with MVX [59]. Only Boston et al. included a control group of dogs treated with surgery and no systemic adjunctive therapy (98 dogs), but included only twelve dogs treated with RT alone [37]. The overall level of evidence for RT therapy alone was a C. Total planned RT delivered ranged from 24 to 48 Gy, and the fraction dosage differed from 4 to 7 Gy/treatment and number of treatments from 3 to 12. Dogs with metastatic disease were not included in any of these studies.

Overall response in two studies ranged between 83% and 94%, although the MST for the dogs that showed an OR of 94% was short (147 days) [57,58]. Theon et al. reported a good MPFI based on tumor stage [59]. Although MDFI cannot be substituted for median survival time, it does give an impression of duration of effectiveness of therapy based on the tumor stage. Overall response was markedly higher than in studies utilizing CT alone [41,42,45].

MST for dogs receiving RT alone (147–307 days) [50,57,58], was similar to RT with adjunctive CT (171–363 days) [52,53], and appeared to result in a longer MST compared to CT alone (116–184 days) [35,42]. The exception to these MSTs was the Boston et al. that showed a MST of 1747 days for RT only compared to 335 with surgery alone [37]. Among dogs that received RT alone in that study, the effect of radiotherapy was confounded by age, with the dogs being younger. The association of RT and increased survival time was not significant in multivariate analysis.

Two studies reported adverse reactions [57,58]. These reactions were only well described by Bateman et al. with mainly low-grade cutaneous reactions with two possible late RT complications, that may not have been related to the RT [58].

Improved survival beyond palliative care with minimal adverse reactions is likely with RT, but due to the lack of uniform RT protocols and reporting of clinical outcomes, further conclusions based on the available studies are not possible. RT alone is best suited to local control with good OR, with RT alone showing only a longer MST compared to CT alone. Further studies are required to investigate if higher total Gy delivered in small fractions is superior to lower Gy delivered in smaller fractions.

### 3.5. Electrochemotherapy

Electrochemotherapy (ECT) combines the administration of a poorly permeant cytotoxic agent with the local application of electric pulses that induce reversible electropolation, thus improving drug diffusion into the cells, providing good local tumor control [60].

One large-scale prospective clinical series (67 dogs) [22] and a smaller prospective case series (≤10 dogs) [61] evaluated ECT in dogs with OMM, but did not include a control group or compare it to an alternative therapy (Table 6). The larger study included only dogs that were not candidates for first-line therapy such as surgery. In a smaller study, Maglietti et al. evaluated the use of ECT with bleomycin in a variety of tumors (including six dogs with OMM) comparing only systemic administration to systemic and intratumoral (IT) administration, in dogs that had failed to achieve a complete response after one ECT session [62]. The remaining two studies were case reports [63,64]. The overall LOE for studies evaluating ECT was C.

The electric pulse, frequency delivered and type of needle electrode used varied between studies. Tellado et al. based the number of ECT sessions on whether it was deemed necessary based on recurrence of the primary tumor [22]. A total of 61% of the patients (41) required a single session, 30% (20) required two, 7% (5) required three and 1% (1) required four procedures. Local response was determined after a median of 1.5 treatments. In contrast, Spugnini et al. delivered a standard four sessions and evaluated the response after the last session [61]. Bleomycin was consistently used for all studies as the cytotoxic agent with all studies administering electropolation 8 min after intravenous administration with the exception of Spugnini et al. in which electropolation was administered five minutes after intravenous administration of bleomycin. Three of the five studies also gave IT bleomycin [61,62,63].

Overall response in the two larger studies was 70–80% [22,61]. The lower response seen in Tellado et al. may have been due to the lack of IT administration of bleomycin, but could also have been due to the higher proportion of dogs in stage III and IV, compared to Spugnini et al. [22]. A complete response was reported in all three dogs given both systemic and local bleomycin compared to partial response (2) and stable disease (1) in dogs given only systemic bleomycin [62].

Median survival time in the Tellado et al. was only reported according to stage, making comparison to other treatment modalities difficult. As would be expected, the MST decreased as the stages increased [22]. A MST of 180 days was reported in the second largest study [61], while the two case reports had shorter survival times than compared to the same WHO stage in the Tellado et al. study [22], but were euthanized for reasons unrelated to OMM and ECT administration [63,64]. The MST achieved with ECT seem comparable to RT therapy, with or without CT, but further studies evaluating MST with ECT and OMM are required.

Adverse side effects following ECT were minor and included bleeding, inflammation and necrosis of the tumor, difficulty eating, tongue edema and mucosal discoloration at the tumor site [22,61,62,63,64].

Electrochemotherapy seems a reasonable option in dogs that are not candidates for surgery or when surgery is declined, and the lack of significant side effects reported for OMM support the therapy as a viable option. The OR is similar to RT, but further studies are required to determine which therapy would afford the longest MST. Although the ideal ECT protocol requires further investigation, systemic and local bleomycin should be considered in all cases.

### 3.6. Hyperthermia

Hyperthermia (HT) is a therapy during which body tissue is heated to as high as 44 °C to damage and ultimately kill cancer cells with little or no harm to surrounding normal tissue. Hyperthermia is not a widely used treatment modality in veterinary medicine due to the need for specialized equipment. Five prospective clinical studies report the use of hyperthermia for the local control of OMM (Table 7) [65,66,67,68,69]. The largest study, a prospective randomized controlled clinical trial consisting of 29 dogs evaluated combined RT and HT compared to RT alone [65]. The remaining studies consisted of four cases or less and were not specifically designed to evaluate OMM, but rather the response across a number of different tumor types and locations [66,67,68,69]. Adjunctive RT was included in three studies [65,66,68], intratumoral cisplatin in another [67] and one case report without the addition of RT or CT [69]. Only two of the smaller studies reported the WHO stage of melanoma [67,68].

Dewhirst et al. showed a greater number of dogs with a CR when RT was combined with HT (80%) compared to RT alone (18%) [65], although the 18% CR reported was markedly lower than those reported by Blackwood et al. (69%) and Bateman et al. (53%), in which RT alone was delivered at lower total doses [57,58]. Additionally, Dewhirst et al. showed evidence for significant improvement in long-term control without an increase in the potential for metastatic spread, but dogs with RT alone had a longer MST than with combination HT and RT therapy [65]. Thompson et al. showed a complete response in ¾ OMM, but no survival times were reported [66]. Stable or progressive disease was noted in all three dogs that underwent hyperthermia and intralesional cisplatin, with survival times for the 3 dogs reported as varying from 14 to 168 days [67].

Adverse events, when reported, were minor and self-resolving, commonly including erythema, mucositis and alopecia [66,67,68].

With the limited available information, HT in combination with RT appears effective for the control of local disease but did not result in longer MST than RT alone. It is likely that RT therapy alone can achieve the same or better outcomes [65]. Further prospective studies with larger numbers are required to further evaluate if HT in combination with RT is more effective than RT alone.

### 3.7. Alternative Therapy

Lupeol is a triterpene found in certain fruits, vegetables and several medicinal plants [70]. Melanoma cell proliferation inhibition has been reported in vitro and in vivo due to lupeol’s antitumor properties [71,72,73]. Melanoma tumor growth was inhibited by systemic lupeol in a mouse model [71].

One prospective case series (OEG C, Table 8) investigating lupeol included eleven dogs that had partial resection and one dog that had complete resection of their tumors [74]. Lupeol was subsequently given subcutaneously at tapering doses for several months. Three dogs received additional adjunctive therapy. At the end of the experimental period, all the dogs remained alive. Of the 12 dogs, 10 had survived for >180 days after surgery. Moreover, only two dogs had local recurrence, and no distant metastasis was observed during the experimental period. No adverse events were noted.

No other studies have evaluated Lupeol in canine tumors, but the results from this one study appear promising and lupeol requires further investigation to determine efficacy against OMM and possibly other canine tumors.

One prospective case series (LOE 4) evaluating the effect of liposome-encapsulated curcumin for naturally occurring canine cancers showed that the only patient with OMM treated had progressive disease [75].

### 3.8. Immunotherapy

The immune system actively attempts to prevent the development of tumors, a process known as ‘cancer immunosurveillance’ [76]. Its ability to recognize and eliminate cancer cells forms the fundamental rationale behind immunotherapy [77], with the focus of the various immunotherapy strategies available being either to stimulate an antitumoral immune response or to minimize the immunosuppressive nature of the tumor microenvironment [78].

#### 3.8.1. Vaccination

Therapeutic vaccination serves to educate the immune system to recognize tumor-specific antigens [79]. Whole-cell tumor vaccines and deoxyribonucleic acid (DNA) vaccines represent the most widely adopted vaccination strategies available for the treatment of canine OMM. Whole-cell tumor vaccines consist of irradiated or lysed tumor cells, with or without immunostimulatory cytokines, and stimulate an immune response against many tumor antigens [80]. While DNA vaccination, mostly bacterial plasmid based and rarely dendritic cell based, encode tumor-specific xenoantigens and elicit antigen-specific humoral and cellular immunity [79].

Oncept™ (Merial, Duluth, GA, USA), a bacterial plasmid DNA vaccine encoding the tumor-targeted antigen human tyrosinase (huTYR pDNA), was the first cancer vaccine to receive full approval from the US Department of Agriculture and is the most extensively investigated vaccine used in dogs with OMM [37,81,82,83,84,85,86].

Case series comprise the body of literature in support of Oncept’s™ role in improving survival times in dogs with OMM in the surgical adjunctive setting (Table 9) [81,82,83,85,86]. Studies incorporating additional treatment modalities, such as RT, CT or tyrosine kinase inhibitors, reported MSTs of 335–455 days in dogs with stage I–III disease [81,85,86]. Adjunctive RT was shown to confer protection against tumor progression in the largest of these studies involving 131 dogs [85]. Only two studies have compared Oncept’s™ efficacy, as a surgery adjunctive, to appropriate control groups and found it to provide no survival advantage [37,84], with MSTs in dogs that received adjunctive vaccination ranging from 335 to 485 days compared to surgical controls with MSTs of 352–585 days [37,84]. The presence of confounding adjunctive treatments, in addition to surgery, as well as the use of another DNA vaccine with unknown constituents (Wisconsin vaccine) in the Boston et al. 2014 study, does, however, complicate the interpretation of these findings [37,84]. Those in which vaccination was evaluated as the sole surgical adjunctive were without control groups and largely involved stage II OMM patients [82,83]. In one of these studies, the MST of stage II vaccinates was not reached by the time of last data analysis, with the lower 25th percentile of survival time in all vaccinates being reported as 464 days [83]. The other, reporting a MST of 806 days in the six dogs still alive at the end of the study period and 357 days in the sixteen that died of progressive disease during the course of the study [82].

The use of dendritic cell-based DNA vaccines has only been described in case reports [87]. Administration of autologous bone marrow derived dendritic cells, expressing xenogeneic human melanoma antigen gp100 (BM-DC Adhgp100), to patients with stage I OMM has shown conflicting results with one patient showing no evidence of disease recurrence at 1440 days and the other a survival time of 210 days [87]. One stage III patient that received treatment survived until drowning at 660 days, with no postmortem examination being conducted to evaluate for presence of disease [87].

Combining whole-cell and DNA vaccination strategies, using an allogenic whole-cell tumor vaccine expressing xenogeneic human glycoprotein 100 (Hgp100-ATCV), resulted in a response rate of only 16% (4/25) and a MST of only 153 days (responders 417 days vs. non-responders 95 days) in a case series of dogs with mainly advanced stage disease [88].

The current available literature lacks sufficient high-quality evidence to support the use of vaccination in dogs with OMM in the surgical adjunctive setting, with the OEG being a C. The survival benefit conferred by vaccination is confounded by a lack of consistent comparative control groups and confounding adjunctive treatment modalities [37,81,82,83,84,85,86]. Randomized, double-blinded, controlled clinical studies evaluating the utility of melanoma vaccines in the surgical adjunctive setting as well as their possible role beyond that of just a surgical adjunctive are warranted.

**Table 9:** Summary of vaccination studies evaluating effectivity in oral malignant melanoma.

Overall Evidence Grade: C											
Immunotherapeutic Agent Evaluated	Primary Treatment	Adjunctive Therapy	Study Type	Number of Dogs	WHO Stage of Melanoma	Response Rate	Median Survival Time (Range) in Days	Median DFI (Range) in Days	Adverse Events	Reference	LOE
ONCEPT™ melanoma vaccine	Not reported	Surgery and/or RT	RCS	131	I: 25 (19%) II: 28 (22%) III: 37 (28%) Unknown: 41 (31%)	OR: 28/37 (76%) CR: 11/37 (30%) PR 17/37 (46%) SD: 9/37 (24%)	442 (352–663)	NE	Local: Hematoma at injection site: 1 Systemic: Lethargy and coughing: 1	[85]	4a
ONCEPT™ melanoma vaccine	Surgery	RT and/or CT and/or other (NSAIDs and Toceranib)	RCS	69	I: 18 (26%) II: 25 (36%) III:23 (33%) IV: 3 (5%)	OR: 4/13 (31%) CR: 3/13 (23%) PR: 1/13 (8%) SD: 3/13 (23%) PD: 6/13 (46%)	I: NR II: 269 (118–421) II: 342 (214–470) IV: 178 I–III: 455 (324–586)	I–III: 222 (175–269)	Local: Pain at injection site: 4 Local erythema: 2 Hair discoloration: 2 SC hemorrhage: 1 Systemic: Lethargy: 2 Lethargy and anorexia: 1 SCC at injection site: 1	[86]	4a
ONCEPT™ melanoma vaccine	Surgery	No	PCS	58	II: 44 (76%) III: 14 (24%)	NE	II: NR III: 235	NE	Local: wheal, hematoma, pain, bruising. Systemic: none	[83]	4a
ONCEPT™ melanoma vaccine	Surgery alone or surgery and RT	RT and/or surgery and/or MCT and/or tyrosine kinase inhibitor and/or CT	RCCS	Total: 45 Study: 22 Control: 23	I: 10 (22%) II: 22 (49%) III: 8 (18%) Unknown: 5 (11%)	NE	II and III: Study: 477 Control: 491 I–III: Study: 485 Control: 585	I and II: Study: 140 Control: 331 I–III: Study: 171 Control: 258	None	[84]	3b
ONCEPT™ melanoma vaccine	Surgery	RT	RCS	32	I: 9 (28%) II: 17 (53%) III: 6 (19%)	NE	All: 335 (301–540) I: 373 (163–913) II: 383 (60–1078) III: 189 (60–428)	NE	None	[81]	4b
ONCEPT™ melanoma vaccine	Surgery	No	RCS	25	II: 23 (92%) III: 1 (4%) IV: 1 (4%)	NE	Alive at end of study (6/25): 806 Died of progressive disease (16/25): 357	NE	None	[82]	4b
ONCEPT™ melanoma vaccine or Wisconsin vaccine	Surgery	CT: 32 Carboplatin (21) platinum-based treatment (5) lomustine (1) dacarbizine (1) doxorubicin (1) and metronomic chemotherapy (4) OR RT:12 Protocol varied but most often hypo- fractionated protocol	RCCS	Study: 24 Oncept: 14 dogs Wisconsin vaccine: 10 dogs Control: 98	NE	NE	Study: 335 Control: 352	NE	NE	[37]	3b
Hgp100-ATCV vaccine	Not reported	No	PCS	25	II: 9 (36%) III: 6 (24%) IV: 10 (40%)	OR: 4/25 (16%) CR: 1/25 (4%) PR: 3/25 (12%) SD: 3/25 (12%) PD: 18/25 (72%)	All dogs: 153 Responders: 417 Non-responders: 95	NE	Mild induration and erythema at vaccination site 1 dog— depigmentation of oral tumor and mucosa	[88]	4b
BM-DC Adhgp100 vaccine	Surgery	RT	PCS	3	I: 2 (67%) III: 1 (33%)	I: CR 1/3 at 1440 days	I: 210 III: 660	NE	None	[87]	4c

Abbreviations: Adj.: adjunctive, BM-DC Adhgp100: autologous bone marrow-derived dendritic cells expressing xenogeneic human melanoma antigen gp100, CR: complete response, CT: chemotherapy, Hgp100-ATCV: allogeneic tumor cell vaccine expressing xenogeneic human melanoma antigen gp100, MCT: metronomic chemotherapy, NE: not evaluated, NR: not reached, NSAID: non-steroidal anti-inflammatory drug, OR: overall response, OS: overall survival, PCS: prospective case series, PR: partial response, RCCS: retrospective controlled case study, RCS: retrospective case series, RT: radiotherapy, SC: subcutaneous, and SD: stable disease.

#### 3.8.2. Electrovaccination and Microseeding

The safety and possible potentiating role of novel vaccine delivery systems, namely microseeding and electrovaccination, have also been evaluated in dogs with OMM [89,90,91,92,93]. DNA microseeding makes use of a modified tattooing device to deposit vaccine at a controlled rate into micropunctures created intradermally (Table 10) [89]. In a pilot study including four dogs with OMM, delivery of a xenogeneic human tyrosinase plasmid DNA vaccine via microseeding was found to be well tolerated but the study lacked sufficient power to evaluate true antitumoral effects [89].

Electrovaccination involves the application of an electric field that serves to increase cell membrane permeability thus facilitating the introduction of molecules such as plasmid DNA [90]. Two prospective non-randomized controlled clinical trials, with no additional confounding treatment modalities, have shown electrovaccination with human chondroitin sulfate proteoglycan-4 DNA vaccine (hCSPG4-DNA) to significantly prolong survival in dogs with surgically resected OMM (Table 11) [91,92]. One study reported a MST of 653 days in CSPG4-positive vaccinates compared to 224 days in both CSPG4-positive and -negative surgical controls [91]. The second prospective study reported CSPG4-positive vaccinates and surgical controls as having a MST of 684 days and 220 days and lung metastatic rates of 39% and 79%, respectively, with dogs less than 20 kg reported as having improved survival times [92]. The extent of surgical excision was shown to significantly influence disease progression in a case series of CSPG4-DNA electro-vaccinated dogs also receiving other therapeutic adjunctives, with a MST of 1333 days reported in dogs undergoing curative intent surgery compared to 470 days in those undergoing only marginal excision [93].

Good-quality evidence supports the use of electrovaccination with hCSPG4-DNA in dogs with OMM, with the OEG for electrovaccination being a B. High-quality studies, with larger case numbers are, however, needed to support the findings of these existing studies.

#### 3.8.3. Gene Therapy

Viral or non-viral vectors, namely liposome delivery or DNA protein complexes, can be used to facilitate the delivery of foreign DNA into cells, a process known as transfection [79]. Local delivery of various gene products results in unique antitumoral responses while limiting the potential toxicity associated with systemic exposure [79,94].

The combined intratumoral administration of suicide gene therapy, where infection of cells with the herpes simplex virus thymidine kinase gene facilitates the activation of ganciclovir, and xenogeneic cells secreting cytokines, specifically human granulocyte–macrophage colony-stimulating factor (hGM-CSF) and interleukin-2 (hIL-2), was shown to significantly delay or prevent distant metastasis and extend survival times in a prospective non-randomized clinical trial, in which 95% of the enrolled melanoma bearing dogs had OMM (Table 12) [95]. With the percentage of metastasis-free patients at study end in the combined treatment group (76%) being significantly higher than the untreated controls (29%), surgery-treated controls (48%) and the suicide gene-treated only controls (56%) [95]. Both the MST and the metastasis-free survival were significantly extended in the combined treatment group (160 days; >509 days, respectively) as compared to untreated controls (69 days; 41 days), surgery-treated controls (82 days; 133 days) and suicide gene-treated only controls (94 days; >159 days) [95]. The overall response rate for the combined treatment group reached 46%, with complete remission of pulmonary metastasis being achieved in one dog that was later euthanized due to primary tumor progression at 123 days post-treatment initiation [95]. This study showed that repeated injections of the suicide gene system and cytokine-secreting xenogeneic cells into the tumor bed could substantially control tumor growth, delay or prevent distant metastasis and significantly extend survival rate [95].

In a prospective non-randomized clinical trial, in which 82% of the enrolled population had OMM, peri-tumoral injection of xenogeneic Vero cells expressing human interleukin-2 as an adjunctive to surgery or RT resulted in a MST of 270 days as compared to 72 days in those receiving only the primary treatment modality [96].

The remaining literature specific to gene therapy is of a low LOE and is presented in the form of case reports and small case series. Immunogene injections encoding potent T-cell activators, namely adenovector CD40 ligand (AdCD40L) or staphylococcal enterotoxin B and canine GM-CSF, or apoptosis promoter, Fas-ligand (FasL) have proved to be safe and resulted in promising antitumoral effects with reported ORs ranging between 55% and 100%, such findings warranting further research [97,98,99,100].

Although these study populations largely consisted of dogs with the oral form of MM, higher-quality studies specific to OMM or at least with more targeted analysis of the oral subpopulation are needed in order for meaningful conclusions to be reached. The OEG for gene therapy is a C.

Electrogene therapy and electrochemogene therapy in dogs with OMM are represented by an OEG of D and C, respectively. Only case reports and small case series detailing the safety and vague antitumoral effects of interleukin-12 (canine-cIL-12 pDNA, feline-fIL-12 pDNA and human-hIL-12 pDNA) electrogene therapy and additional bleomycin therapy are available [101,102,103,104]. Further studies are necessary.

**Table 12:** Summary of gene therapy studies evaluating effectivity in oral malignant melanoma.

Overall Evidence Grade: C											
Immunotherapeutic Agent Evaluated	Primary Treatment	Adjunctive Therapy	Study Type	Number of Dogs	WHO Stage of Melanoma	Response Rate	Median Survival Time (Range) in Days	Metastasis-Free Survival (Range) in Days	Adverse Events	Reference	LOE
Lipid-complexed herpes simplex thymidine kinase with ganciclovir (suicide gene therapy) alone (SG) or with irradiated transgenic xenogeneic cells secreting hGM-CSF and hIL-2 (CT)	NE	No	PNRCT Results not specific to OMM. OMM > 80% of cases	101 UC: 17 SC: 23 SG: 16 CT: 45	I & II: 22 (22%) III: 67 (66%) IV: 12 (12%)	SG: OR: 7/16 (44%) PR: 6/16 (38%) CR: 1/16 (6%) SD: 4/16 (25%) PD: 5/16 (31%) CT: OR: 21/45 (46%) PR: 14/45 (31%) CR: 7/45 (15%) SD: 12/45 (27%) PD: 12/45 (27%)	UC: 69 (10–169) SC: 82 (43–2160) SG: 94 (46–159) CT: 160 (57–509)	UC: 41 (10–169) SC: 133 (43–216) SG: >159 (41–159) CT: >509 (57–509)	At Injection site: De- pigmentation (20%) Edema (30%) Swelling & itching (17%)	[95]	3b
Xenogenic Vero cells secreting hIL-2	Surgery and RT	No	PNRCT Results not specific to OMM. OMM> 80% of cases	32 Study: 16 Control: 16	NE	NE	Study: 270 Control: 72	NE	Local inflammation at injection site	[96]	3b
Intratumoral of *Staphylococcal* enterotoxin B DNA	NE	No	PCS Results not specific to OMM. OMM> 80% of cases	Total: 22 OMM: 20	I: 3 (14%) II: 5 (23%) III: 12 (54%) IV: 2 (9%)	OR: I–IV: 12/22 (55%) I: 100% II: 60% III: 33% IV: 0%	I: 427 II: 399 III: 168 IV: 0	NE	Transient peri- tumoral edema after injection: 1 Transient anorexia: 1	[99]	4b
Intratumoral AdCD40L	NE	Surgery or chemotherapy	PCS	14	I: 2 (14%) II: 1 (7%) III: 8 (57%) IV: 3 (22%) Specific to OMM	OR: 11/14 (79%) CR: 4/14 (29%) PR: 7/14 (50%) SD: 3/14 (21%) Specific to OMM	I–IV: 160 (20–1141) II-IV: 131 Not specific to OMM	NE	Mild transient fever: 7 Mild anorexia: 5 Injection site swelling: 3 Mild transient liver enzyme elevation:2 Not specific to OMM	[97]	4c
Intratumoral FasL DNA	NE	Surgery and/or RT	PCS	4	III: 4 (100%)	OR: 4/4 (100%) CR: 2/4 (50%) PR: 2/4 (50%)	(91–574)	NE	None	[100]	4c
Intratumoral human AdCD40L	Local dia- thermia	CRS	Case report	1	IV: 1 (100%)	CR: 1 (100%)	401	NE	Mild swelling of tumor	[98]	4d
Electrogene therapy											
Intratumoral hIL-12 pDNA with EP	NE	MCT (cyclophosphamide)	Case report	1	IV: 1 (100%)	PD	NE	NE	Local swelling & erythema 2–3 days later	[101]	4d
Intratumoral hIL-12 pDNA with EP	NE	No	Case report	1	IV: 1 (100%)	Regression- progression cycles 1^st^ Tx: PR 2^nd^ Tx: PR 3^rd^ Tx: PD 4^th^ Tx: N/A 5^th^ Tx: SD	NE	NE	None	[102]	4d
Electrochemogene therapy											
cIL-12 pDNA ECT & IV bleomycin	NE	CRS	PCS	9	I: 2 II: 4 III: 3	OR: 6/9 (67%)	180	NE	Transient leucocytosis & neutrophilia: 4	[104]	4c
fIL-12 pDNA ECT & Intralesional bleomycin	NE	Piroxicam & tramadol	Case report	1	IV: 1	PR: 1	NE	NE	48 hours of diarrhea	[103]	4d

Abbreviations: AdCD40L: adenovector CD40 ligand, cIL: canine interleukin, CR: complete response, CRS: cytoreductive surgery, CT: combined therapy group, DNA: deoxyribonucleic acid, ECT: electrochemotherapy; EP: electroporation, FasL: Fas ligand, fIL: feline interleukin, GM-CSF: granulocyte-macrophage colony-stimulating factor, hGM-CSF: human granulocyte–macrophage colony-stimulating factor, hIL: human interleukin, IV: intravenous, MCT: metronomic chemotherapy, N/A: not applicable, NE: not evaluated, OR: overall response, PCS: prospective case series, PD: progressive disease, pDNA: plasmid deoxyribonucleic acid, PNRCT: prospective non-randomized controlled clinical trial, PR: partial response, RT: radiotherapy, SC: surgical control group, SD: stable disease, SG: suicide gene treated group, Tx: treatment, UC: untreated control group, and US: ultrasound.

#### 3.8.4. Combination of Gene Therapy and Vaccination

High-quality studies investigated combination gene therapy and vaccination in dogs with OMM (Table 13). Two, large, prospective non-randomized clinical trials, each incorporating over 400 dogs of varying metastatic stage, receiving no additional confounding therapeutics, evaluated a combined immunotherapy strategy in the surgical adjunctive setting [105,106]. In both studies, suicide gene therapy (lipid-complexed thymidine kinase suicide gene in combination with ganciclovir) and plasmid DNA-encoding canine interferon-beta (cIFNβ), with the addition of local bleomycin therapy in the second study, was co-injected with an autologous, tumor cell, cytokine-enhanced (hIL-2 and hGM-CSF) vaccine [105,106].

In the first study, long-term follow up, six years post-treatment, showed this combination therapy to be safe and capable of delaying or preventing post-surgical recurrence and distant metastasis [105]. Adjunctive combination therapy was shown to increase MST in dogs undergoing complete surgical resection of their primary tumors from 101 days to 704 days, their median DFI from 62 days to >2251 days, the proportion of local disease-free patients from 11% to 83% and distant metastasis-free patients from 44% to 89% [105]. Even in dogs undergoing partial resection of their primary tumor, adjunctive combination immunotherapy increased MST from 78 days to 323 days and increased the proportion of metastasis-free patients from 48% to 82% [105].

The second study highlighted the added benefit of combination therapy, compared to autologous tumor vaccine therapy alone, as an adjunctive to surgery [106]. Median survival times in dogs undergoing complete surgical resection of their primary tumor were increased from 95 days to 614 days in vaccinates and 880 days in those receiving combination therapy [106]. The proportion of local disease-free patients was increased from 20% in surgical controls undergoing complete resection to 74% in those receiving adjunctive vaccination and 89% in those receiving combination therapy, with the proportion of distant metastasis-free patients being increased to 84% in vaccinates and 87% in combined therapy patients from 45% in surgical controls undergoing complete resection [106]. As demonstrated in the previous study, adjunctive combination immunotherapy was beneficial even in dogs undergoing partial resection of their primary tumor, increasing the percentage of distant metastasis-free patients from 44% to 80% [106].

It is proposed that this combined immunotherapy strategy is capable of altering the course of a once lethal disease, supported by the fact that 1% of dogs undergoing complete surgical resection of their primary tumors died of melanoma-unrelated causes compared to 51% who received adjunctive vaccination and 70% receiving combined immunotherapy [106]. The OEG of a combined immunotherapy strategy incorporating both vaccination and gene therapy is a B. This combined immunotherapy strategy compromises the highest level of evidence available in support of an immunological surgical adjunctive for extending survival in dogs with OMM. Further prospective randomized, double-blinded, controlled clinical trials are warranted.

#### 3.8.5. Checkpoint Inhibitors

T lymphocytes are the primary effector cells involved in the adaptive immune response against tumors [79]. Immune checkpoints are surface receptors on T lymphocytes that provide regulatory feedback to limit the effector phase of T-cell expansion and function [79]. Immune checkpoints play a key role in the development of tolerance to self-antigens in health but their upregulation in many tumors plays a critical role in tumor-associated immune suppression and evasion [79].

Inhibiting tumor-associated immunosuppression and thus enhancing autoimmunity can be achieved through the targeting of inhibitory immune checkpoints using monoclonal antibodies [79,107]. A chimeric anti-programmed cell death ligand 1 (PD-L1) monoclonal antibody (c4G12) has been shown in a prospective non-randomized clinical trial to improve survival in dogs with stage IV disease, with a MST of 143 days being achieved in the treatment group compared to 54 days in the institutional historical control group (Table 14) [108]. Its antitumoral response, however, is less apparent in another retrospective study, with an OR rate of just 14% (1/7) and no statistically significant change in MST in dogs with stage IV disease being achieved [108,109]. A MST of 166 days compared to 55 days in institutional historical controls was reported in a prospective non-randomized clinical trials evaluating variable (chimeric rat-dog-ch-4F12-E6 and caninized-ca-4F12-E6) anti-programmed cell death protein 1 (PD-1) monoclonal antibodies in dogs with late-stage disease (91% stage IV) [108,110]. Adjunctive therapies do, however, unfortunately complicate the applicability of the findings from these studies [108,109,110], with adjunctive RT being shown to improve the overall survival of the treatment group in one [108]. Another monoclonal antibody, chimeric mouse-dog anti-podoplanin (PDPN)-P388f, has been investigated in a small case series involving three dogs, but the focus of this study was on the treatment’s safety profile rather than antitumoral effects [111]. The OEG for checkpoint inhibitors is a B, although sufficient good-quality evidence supporting the use of checkpoint inhibitors in dogs with OMM, in the surgical adjunctive setting, is lacking. Well-designed studies with less confounding elements and investigation into the utility of this treatment modality in less advanced stages of disease are warranted.

#### 3.8.6. Bacteria

In the infancy of immunotherapies, bacteria, particularly *Corynebacterium parvum,* was injected intratumorally in an attempt to stimulate an antitumoral immune response (Table 15). A non-randomized, well-designed study, involving 89 dogs (stage I–III) evenly split between treatment and control groups, showed the capability of this adjunctive treatment to prolong survival, with a MST of 370 days reported in treated patients as compared to 228 days in surgical controls [36]. A case series describing the same treatment, published at a similar time, found it to be unsuccessful in dogs with gingival melanoma, with all dogs dying due to disease progression within six months of treatment [112]. The OEG for this modality is a B. Minimal, dated literature and a focus on advances in more current immunotherapy strategies have resulted in this strategy largely falling out of favor.

#### 3.8.7. Stimulatory Cytokines

The only prospective, randomized, double-blinded controlled trial investigating immunotherapy in dogs with OMM evaluated the efficacy of systemic administration of liposome encapsulated muramyl tripeptide-phosphatidylethanolamine (L-MTP-PE) alone or in combination with GM-CSF in the surgical adjunctive setting (Table 16). Whether used alone or in combination with GM-CSF, L-MTP-PE was found to have a minimal antitumoral effect in dogs with advanced stage disease. However, in dogs with early, stage I disease, it was shown to prolong survival time with the MST not being reached in treated patients as compared to 414 days in the surgical controls [113].

A recent retrospective non-randomized clinical trial has demonstrated the ability of combination therapy with interferon-alpha and carboplatin to increase the overall survival times in dogs when used in the surgical adjunctive setting [114], with a MST as high as 894 days being achieved in the 17 dogs undergoing treatment as compared to 86 days in the 3 surgical controls [114].

A small case series describing the systemic administration of recombinant human tumor necrosis factor (rhTNF) and recombinant human interleukin 2 (rhIL-2), in combination with a variety of other adjunctive treatments, reported tumor regression in 39% of treated patients (OR 38%), with one death being reported after TNF administration [115].

The OEG for the use of stimulatory cytokines as surgical adjunctives is a B. Currently there is only sufficient evidence to support their use, in dogs with stage I disease [113]. Recent findings do, however, suggest that interferon-alpha in combination with carboplatin could show promise as a surgical adjunctive and warrants further investigation in a prospective setting [114].

#### 3.8.8. Nanotechnology

Low-quality evidence, in the form of case reports without consideration for clinical stage, attempt to describe the additional immunostimulatory benefit conferred by ‘in situ’ vaccination with nanoparticles to traditional RT and hyperthermia treatment (Table 17) [116,117]. Survival times of up to 780 and 1350 days were reported in dogs receiving magnetic iron oxide nanoparticle hyperthermia (mNPH) treatment in isolation, remission at 600 days when plant-based virus-like nanoparticles (VLP) treatment was used in combination with RT and remission at 300 and 540 days in dogs undergoing a combination of all three therapies [116,117]. The use of nanotechnology in dogs with OMM lacks good-quality supporting evidence with an OEG of D.

#### 3.8.9. Immunotherapy Conclusions and Future

Adjunctive immunotherapy shows promise for a significant survival advantage in dogs with oral malignant melanoma as compared to previous standards of care, namely: surgery, CT and RT, when used in isolation [107]. This most evident in combination gene therapy and vaccination’s ability to increase MST from 95–101 days to 704–880 days in dogs undergoing complete surgical resection of their primary tumors, with 70% of dogs undergoing adjunctive combination therapy dying of melanoma-unrelated causes compared to 1% in those undergoing surgical resection of their tumor in isolation [105,106].

Although immuno-oncology currently offers the most targeted, precise approach to cancer therapy, this field is still in its infancy and future advances with regard to predictors of response will allow for further tailoring of treatments to the factors that make each tumor and host a unique pairing [76,107].

## 4. Conclusions

Numerous factors affected the interpretation of the studies presented above; of particular importance was the classification and staging of OMM, the presence of confounding treatment modalities, as well as a lack of randomization, control groups, consistent reporting of prognostic indicators, treatment protocols and clinical outcomes. The current adoption of the WHO classification for melanocytic tumors may be problematic as the current WHO classification system was not seen as appropriate to determine prognosis based on clinical stage of OMM [38]. An alternate staging system was proposed that included tumor volume rather than diameter, tumor location and mitotic index, as these were found to be prognostic in a study of forty-one dogs [38]. This system has, however, not been accepted universally as no studies reviewed used this system. In the human literature, melanomas of the oral cavity (mucosal melanomas) have their own classification system (mmTNM), separate from skin melanomas, as mucosal melanomas are so much more aggressive [118,119]. Currently, identified prognostic indicators in dogs include stage of disease, presence of distant metastasis, nuclear atypia, mitotic index, degree of pigmentation, lymphatic invasion and Ki67 index [120]. Many of the studies evaluated here were published before these prognostic indicators were identified and were therefore not reported in many of the studies. In future studies evaluating the response to a particular therapy, the identified prognostic indicators should be described to allow comparison between studies. Further to this classification and staging system, the following parameters have been shown to be prognostic in humans and it is advised that studies should report these additional features: ulceration, tumor infiltrating lymphocytes and melanoma subtype [121].

The mitotic index has been identified as a prognostic indicator in humans and dogs [120,121]. A mitotic index of 3 or less per 10 high-power fields was among the defining criteria for the HWDMN group of melanocytic tumors, together with being highly pigmented and round to elongated nuclei [18]. Future studies should elucidate if these are indeed a different subset of mucosal melanocytic tumors as this would affect prognostic indicators and survival times.

Complete excision and local adjunctive therapy did not prevent metastasis, with studies reporting metastasis even in the event of histologically “clean” margins [26,34,38]. Metastatic disease is an important variable associated with survival, as is control of local disease [26,31,34,38]. The evaluation of histologic margins is essential for the selection of appropriate treatment for the local control of OMM. Terminology such as clean, dirty, close or narrow surgical margins leads to inconsistency in reporting and recently a standardized margin assessment was suggested [120,122]. The residual tumor classification scheme requires the pathologist to report the actual distance in millimeters from the inked surgical edge to tumor cells. A surgical clean margin is described where the distance from inked surgical margin to tumor cells is greater than 0 mm [122]. The advantage of such a system would be to investigate the benefits of a clean margin on the prognosis and allow easier comparison of adjunctive therapy studies in future [122].

Reporting guidelines as suggested by the Recommended Guidelines for the Conduct and Evaluation of Prognostic Studies in Veterinary Oncology were not adhered to by the studies evaluated here, but many of the studies were published before the establishment of the guidelines [21]. Established guidelines for the reporting of treatment response or prognosis should be adhered to in order to allow for appropriate evaluation of data. Additionally, many studies lacked control groups, making the evaluation of the efficacy of the adjunctive therapy difficult.

Based on the information and grade of evidence in this critical review, surgery remains the mainstay of therapy, with no difference in the MST if the tumors are restricted to the oral mucosa, invading bone or are only localized to the tongue. Throughout the reported studies, no comparable criteria were used for location of the tumor, making the evaluation of location as a prognostic indicator very difficult. Adjunctive RT provides good local control and longer MSTs compared to CT alone. Addition of CT to radiotherapy did not appear to improve survival times. Electrochemotherapy seems a reasonable alternative to RT, but further studies are required. In the relatively newer field of immunotherapy, electrovaccination and in particular the combination of gene therapy and vaccination appear to be the most promising in prolonging survival time in the surgical adjunctive setting, but further prospective, double-blinded, randomized, controlled studies without confounding adjunctive therapies are required.

## Figures and Tables

**Table 1:** Format used to grade (**a**) individual references and (**b**) overall level of evidence. Adapted from Elwood and others 2010 [23].

**(a) Study Type Level of Evidence (LOE)**	**Level of Evidence**
Systematic review (with homogeneity) of randomized controlled clinical trials (RCT)	1a
Individual RCT (with narrow confidence interval)	1b
All or none	1c
Systematic review (with homogeneity) of cohort studies	2a
Individual cohort study (including low-quality RCT; for, e.g., <80% follow-up) or well-controlled laboratory study	2b
“Outcomes” research; ecological studies	2c
Systematic review (with homogeneity) of case–control studies	3a
Individual case–control study or non-randomized controlled clinical trial/study or weak laboratory study	3b
Case series > 50 cases	4a
Case series 20 to 50 cases	4b
Case series < 20 cases	4c
Case report	4d
Expert opinion without explicit critical appraisal, or based on physiology, bench research or “first principles”	5
**(b) Types of Study**	**Overall Evidence Grade**
Consistent RCT, cohort study, all or none *, decision rule validated in different populations	A
Consistent retrospective cohort, exploratory cohort, ecological study, outcomes research, good laboratory study, case–control study, non-randomized controlled clinical trial/study; or extrapolations from level A studies	B
Case series study or extrapolations from level B studies.	C
Expert opinion without explicit critical appraisal, or based on physiology, bench research or first principles	D

* The all or none principle is met when all patients died before the treatment became available, but some now survive on it.

**Table 5:** Summary of radiotherapy without adjunctive therapy studies evaluating effectivity in oral malignant melanoma.

Overall Evidence Grade: C										
Radiotherapy Treatment Protocol	Initial Surgery	Study Type	Number of Dogs	WHO STAGE of Melanoma	Response Rate	Median PFI in Days	Median Survival Time (Range) in Days	Adverse Events	Reference	LOE
48 Gy given in 12 fractions over 4 weeks (Monday/Wednesday/Friday schedule) at 4 Gy/fraction	Yes No recurrence included	PCS	38	I–III: numbers not specified	NE	237 I: 564 I: 180 III: 201	NE	NE for OMM	[59]	4b
36 Gy in 4 fractions of 9 Gy at 7 day intervals. Ipsilateral lymph nodes radiated in 17 dogs: 18–27 Gy in 2 or 5 fractions	Yes: 24 22 had recurrence	PCS	36	<III: 26 (72%) III: 9 (25%) Not staged: 1 (3%)	OR: 34 (94%) CR: 25 (69%) PR: 9 (25%) SD: 2 (6%)	NE	147 (35–1491)	“Most dogs”: Grd 1 cutaneous Late radiation toxicities (necrosis): 2	[57]	4b
24 Gy in 7 Gy fractions on day 0, 7 and 21	Yes: 11 Only macroscopic disease included	PCS	18	I: 6 (33%) II: 6 (33%) III: 6 (33%)	OR: 14 (83%) CR: 9 (53%) PR: 5 (30%) SD: 3 (17%)	CR Regrowth: 2 (90 and 195) Disease free: 5 (270, 270, 405, 450, 570) Death from metastasis: 1 Death from intercurrent disease: 10	237	Acute cutaneous Grd 1: 12 Grd 2: 5 Late radiation toxicity: Tooth root abscess (5 months later) Chronic sialocele abscessed (3 months later)	[58]	4c

Abbreviations: CR: complete response, Grd: grade, Gy: Gray, NE: not evaluated, PCS: prospective case series, PFI: progression-free interval, OR: overall response, PR: partial response, and SD: stable disease.

**Table 6:** Summary of electrochemotherapy studies evaluating effectivity in oral malignant melanoma.

Overall Evidence Grade: C											
Electrochemotherapy Treatment Protocol	CT Drug and Dosage	Adjunctive Treatment	Study Type	Number of Dogs	WHO Stage of Melanoma	Response Rate	Median PFI (Range) in Days	Median Survival Time (Range) in Days	Adverse Events	Reference	LOE
Eight minutes after IV bleomycin, electropolation (BTX ECM 830) with each train of pulses: eight square wave monopolar pulses of 400 V (1000 V/cm) 100 μs long at 10 Hz. Number of trains applied varied according to tumor size, aiming to cover the whole tumor volume plus safety margins beyond it. A 6-needle electrode used for all cases, but for nasal duct invasion, the single needle electrode^®^ was indicated	Bleomycin, IV, 15,000 IU/m^2^	No	PCS	67	I: 11 (16%) II: 19 (29%) III: 26 (39%) IV: 11 (16%)	* OR: 47 (70%) CR: 14 (21%) PR: 33 (49%) SD: 11 (17%) PD: 9 (13%)	I: 330 (120–900) II: 210 (90–630) III: 120 (60–120) IV: 120 (30–120)	I: 495 (120–900) II: 270 (120–630) III: 225 (90–510) IV: 135 (60–210)	Bleeding, pain, difficulty eating	[22]	4a
Four sessions of ECT, 1 week apart. Five minutes after bleomycin, sequential bursts of eight biphasic pulses lasting 50 + 50 ms were applied at a voltage of 800 V/cm using modified caliper and needle electrodes using a Chemopulse. The pulse repetition frequency was 1 Hz, burst repetition frequency was 1 kHz, total burst duration of 7.1 ms	Bleomycin, IT and peri- tumor (1cm surrounding the tumor at 1.5 IU/mL	Surgery: pre-ECT in six dogs with subsequent local recurrence	PCS	10	II: 6 (60%) III: 4 (40%)	OR: 8 (80%) CR = 7 (70%) SD = 2 (20%) PR = 1 (10%)	NE	180 (CI: 0–514)	Mucosal discoloration at tumor site: 3	[61]	4c
Eight minutes after IV and IT bleomycin, electroporation (BTX ECM 830) pulses were administered using a 6-needle electrode. A train of 8 electric pulses (1000 V/cm, 100 microseconds, 10 Hz) was applied, covering the whole tumor	Bleomycin, IV, 15,000 IU/m^2^ after bleomycin, IT, 125 IU/cm³ of tumor	No	PNRCT	Control: 3 Study: 3	Control: I:1 II: 2 Study: II: 1 III: 2	Control I: SD II: PR (2) Study All: CR (3)	NE	NE	No toxicity or side effects	[62]	3b
At the time of surgery, 8 min after IV and IT bleomycin, electropolation (BTX ECM 830 square wave) using two types of electrodes 1) two-needle-array (BTX model 532), and 2) Petri Pulser Electrode was performed. Additional Rx on day 14 when metastasis detected in ln.: ECT with calcium ions (CaCl2 in low concentration at 5 mM, 10 mL delivered i.t.) performed directly on the metastatic lymph nodes and remaining tumor mass. Only two-needle-array electrodes were used and in each application the electric field was 8 square wave pulses of 100 μs each, delivered at 1 Hz and voltage of 650 V.	Bleomycin, 0.3 mg/kg IV and 3 mg/mL IT	Surgery: Debulking (CO_2_ laser, 0.25 mm spot diameter, in the continuous wave mode	Case report	1	IV: 1	N/A	N/A	60 day (euthanized due to unrelated seizures)	Inflammation and necrosis of tissue that received ECT	[63]	4d
Eight minutes after bleomycin, a 6-needle electrode applied 8 pulses of 1000 V/cm with a length interval of 100 µsec at a repetition frequency of 10 Hz, using a BTX ECM 830	Bleomycin, IV, 15,000 IU/m^2^	No	Case report	1	III	PR	NE	33 (death due to unrelated condition)	Short-lived edema of the tongue	[64]	4d

Abbreviations: CI: confidence interval, CR: complete response, CT: chemotherapy, ECT: electrochemotherapy, IV: intravenous, IT: intratumoral, IU: international units, IV: intravenous, ln.: lymph node, N/A: not applicable, NE: not evaluated, OR: overall response, PCS: prospective case series, PD: progressive disease, PFI: progression-free interval, PNRCT: prospective non-randomized controlled clinical trial, PR: partial response, and SD: stable disease. * Based on a median of 1.5 treatment sessions.

**Table 7:** Summary of hyperthermia studies evaluating effectivity in oral malignant melanoma.

Overall Evidence Grade: C											
Hyperthermia Treatment Protocol	Initial Surgery	Adjunctive Treatment Protocol	Study Type	Number of Dogs	WHO Stage of Melanoma	Response Rate	Median PFI in Days	Median Survival Time in Days	Adverse Events	Reference	LOE
HT: 500 kHz high-frequency current or 2450 MHz microwaves. (1) 30 min at a minimum of 42 °C once weekly, immediately prior to radiotherapy (17) (2) 60 min at a minimum of 42 °C twice weekly, immediately prior to radiotherapy (5) (3) 60 min at a minimum of 42 °C twice weekly, 2–3 h after radiotherapy (1)	Yes, numbers not specified	RT to primary and accessible regional ln.: 36.8 Gy in 8 fractions of 4.6 Gy/fraction, twice weekly	PRCT	29 RT alone: 11 RT & HT: 15	Not specified	RT alone: OR: 11 (100%) CR: 2 (18%) PR: 9 (78%) RT and HT: OR: 15 (100%) CR: 12 (80%) PR: 3 (20%)	NE	RT alone: 262 RT and HT: 165	NE	[65]	2b
1 or 2 treatments within 10–20 min of RT. Water was circulated at 40 °C	No	RT planned protocol: 36–40 Gys in 9–10 Gy fractions at weekly intervals over 4 weeks. Final dose for OMM not specified	PCS	4	Not specified	OR: 4 (100%) CR: 3 (75%) PR: 1 (25%) All showed recurrence	NE	NE	Erythema, mucositis, localized hair loss, tumor necrosis. Not specified for OMM	[66]	4c
Initiated 15 min after intratumoral cisplatin. Tumor goal temperature was 42 °C for 30 min at steady state	Yes, in some. All had post- surgical recurrence	Localized cisplatin therapy, q7 days, 4 consecutive weeks. Cisplatin formulated to deliver to 3.3 mg/mL with a collagen concentration of 32.5 mg/mL. Delivered into tumors until mixture extruded throughout the treatment volume	PCS	3	II: 2 IV: 1	SD or PD: 3	NE	14, 112 and 168	Grd 1: Local erythema: 1 Grd 2: patchy mucositis: 1 Grd 3: Confluent fibrinous mucositis: 1	[67]	4c
Once a week for 3 sessions. Effective heating time: 45 min, from the first intra-tumoral temperature sensor reached ≥41 °C, or after 15 min of heating-up time had elapsed		RT: 32 Gy delivered in 4 × 8 Gy weekly Additionally: Metronomic chemo Temozolomide (2 cycles)	PCS	1	III	PR	178	360	Grd 1: Acute radiation therapy toxicity	[68]	4d
Laser-induced HT: 60-Watt surgical diode laser with a spectral output of 810 nm (±20 nm), directed into a 4-way beamsplitter and launched through four 400-µm diameter quartz microlens fibers. Each tumor was treated with 500 mW/cm^2^ laser energy for 30 min, weekly for 4 treatments	Yes. Post- surgical recurrence	No	PCS	1	Not specified	PR	21	NE	Not reported for OMM	[69]	4d

Abbreviations: CR: complete response, Grd: grade, Gy: Gray, HT: hyperthermia, ln: lymph node, NE: not evaluated, OR: overall response, PCS: prospective case series, PRCT: prospective randomized controlled clinical trial, PFI: progression-free interval, PR: partial response, RT: radiation therapy, SD: stable disease, and Sx: surgery.

**Table 8:** Summary of an alternative study evaluating effectivity in oral malignant melanoma.

Overall Evidence Grade: C												
Drug	Initial Surgery	CT Treatment Protocol	Adjunctive Therapy	Study Type	Number of Dogs	Stage of Melanoma Evaluated	Response Rate	Median DFI in Days	Survival Time in Days	Adverse Events	Reference	LOE
Lupeol	Yes, all dogs	10 mg/kg, SC, 1 week post-operatively. Initially administered twice a week for 2 weeks Then, decreased to once a week for 4 weeks; then alternate weeks for 8 weeks; then once a month for several months (≥2 administrations); finally, discontinued	1 = melphalan and piroxicam 1 = photodynamic hyperthermal CT 1 = photodynamic hyperthermal therapy	PCS	12	I: 3 (25%) II: 5 (42%) III: 3 (25%) Not staged: 1 (8%)	CR: 1/11 (9%) PR: 10/11 (91%)	170	>180 after surgery: 10 All 10 still alive at the end of study	No severe adverse effects	[74]	4c

Abbreviations: CT: chemotherapy, CR: complete response, DFI: disease-free interval, PCS: prospective case series, PR: partial response, and SC: subcutaneous.

**Table 10:** Summary of vaccine microseeding studies evaluating effectivity in oral malignant melanoma.

Overall Evidence Grade: C											
Immunotherapeutic Agent Evaluated	Primary Treatment	Adjunctive Therapy	Study Type	Number of Dogs	WHO Stage of Melanoma	Response Rate	Median PFI in Days	Survival in Days	Adverse Events	Reference	LOE
Xenogeneic human tyrosinase plasmid DNA vaccine with microseeding	Surgery	No	PCS	4	II: 1 (25%) III: 1 (25%) IV: 2 (50%)	CR: 1/4 (25%) PD 3/4 (75%)	II: 412 III: 57 IV: 0	II: 412 + III: 367 IV: 14 and 101	Local irritation at vaccine site within 24 h. Resolved at 2 week f/up.	[89]	4c

Abbreviations: CR: complete response, PCS: prospective case series, and PD: progressive disease.

**Table 11:** Summary of electrovaccination studies evaluating effectivity in oral malignant melanoma.

Overall Evidence Grade: B												
Immunotherapeutic Agent Evaluated	Primary Treatment	Adjunctive Therapy	Study Type	Number of Dogs	WHO Stage of Melanoma	Response Rate	Median DFI (Range) in Days	Median Survival Time (Range) in Days	Outcome (Other)	Adverse Events	Reference	LOE
CSPG4-DNA vaccine with EP	Surgery (Curative intent and marginal excision)	RT and/or metronomic treatment (NSAID/CT) and/or CT and/or ECT	RCS	82 CIS: 51 MES: 31	I–IV not specified for patients receiving vaccine	CIS SD: 19 (37%) PD: 32 (63%) MES SD: 4 (13%) PD: 27 (87%)	CIS: 324 (37–2632) MES: 184 (13-1049)	CIS: 1333 (78–2632) MES: 470 (187-1063)	LRR: CIS: 45.1% MES: 54.8% CM: 51.3% IM: 82.4%	None	[93]	4a
hCSPG4-DNA vaccine with EP	Surgery	No	PNRCT	42 V: 23 NV: 19	V: II: 9 (39%) III: 14 (61%) NV: II: 6 (32%) III: 13 (68%)	NE	V: 477 (50–1694) NV: 180 (38-1250)	V: 684 (78–1694) NV: 220 (75-1507)	LRR: V: 34.8% NV: 42% LM: V: 39% NV: 79%	None	[92]	3b
hCSPG4-DNA vaccine with EP	Surgery	No	PNRCT	33 V: 14 NV: 19	V: II: 5 (36%) III: 9 (64%) NV: II: 5 (26%) III: 14 (74%)	NE	V: 477 (207–∞) NV: 180 (165–∞)	V: 653 (458–∞) NV: 224 (185–∞)	NE	Local: Transient erythema at injection site	[91]	3b

Abbreviations: CSPG4-DNA: chondroitin sulfate proteoglycan-4 deoxyribonucleic acid, CIS: curative intent surgery, CM: clean margins, CT: chemotherapy, ECT: electrochemotherapy, EP: electroporation, hCSPG4-DNA: human chondroitin sulfate proteoglycan-4 deoxyribonucleic acid, huTYR pDNA: xenogeneic human tyrosinase plasmid deoxyribonucleic acid, IM: infiltrated margins, LM: lung metastasis, LRR: local recurrence rate, MES: marginal excision surgery, NE: not evaluated, NSAID: non-steroidal anti-inflammatory drug, NV: non-vaccinates, PD: progressive disease, PNRCS: prospective non-randomized controlled clinical trial, RCS: retrospective case series, RT: radiation therapy, SD: stable disease, and V: vaccinates.

**Table 13:** Summary of vaccination and gene therapy combination studies evaluating effectivity in oral malignant melanoma.

Overall Evidence Grade: B											
Immunotherapeutic Agent Evaluated	Primary Treatment	Adjunctive Therapy	Study Type	Number of Dogs	WHO Stage of Melanoma	Median Survival Time (Range) in Days	Median DFI (Range) in Days	Outcome (Other)	Adverse Events	Reference	LOE
Local cIFNβ and suicide gene therapy (lipid-complexed thymidine kinase suicide gene plus ganciclovir) and Vaccine composed of tumor extracts and lipoplexes carrying hIL-2 and hGM-CSF genes =CT	Surgery	No	PNRCT Results not specific for OMM. OMM > 80% of cases	Total: 464 OMM: 400 S: 163 CS: 98 PS: 65 S-CT: 301 CS-CT: 185 PS-CT: 116	CS: I & II: 41 (42%) III: 51 (52%) IV: 6 (6%) CS-CT: I & II: 75 (40.5%) III: 99 (53.5%) IV: 11 (6%) PS: I & II: 17 (26%) III: 41 (63%) IV: 7 (11%) PS-CT: II & III: 29 (25%) III: 75 (65%) IV: 12 (10%)	CS: 101 (11–568) CS-CT: 704 (99–2251) PS: 78 (29–206) PS-CT: 323 (46–1321)	CS: 62 (8–425) CS-CT: >2251 (69–2251)	Proportion local disease free: CS: 11% CS-CT: 83% Proportion metastasis free: CS: 44% CS-CT: 89% PS: 48% PS-CT: 82%	Induration at injection site (14%) & 24 hours lethargy (22%) Itching & swelling (19%) 12-36 hours after surgery	[105]	3b
Local cIFNβ plus bleomycin and suicide gene therapy (lipid-complexed thymidine kinase suicide gene plus ganciclovir) and (=CT) /or (=V) Vaccine composed of tumor extracts & lipoplexes carrying hIL-2 & hGM-CSF genes	Surgery	No	PNRCT Results not specific to OMM. OMM > 80% of cases	Total: 537 OMM: 439 S: 173 CS: 105 PS: 68 CS-V: 154 CS-CT: 98 PS-CT: 112	CS: I & II: 41 (39%) III: 55 (52%) IV: 9 (9%) CS-V: I & II: 55 (36%) III: 87 (56%) IV: 12 (8%) CS-CT: I & II: 38 (39%) III: 51 (52%) IV: 9 (9%) PS: I & II: 24 (35%) III: 36 (53%) IV: 8 (12%) PS-CT: I & II: 42 (37%) III: 59 (53%) IV: 11 (10%)	CS: 95 (10–540) CS-V: 614 (121–1896) CS-CT: 880 (177–2129) PS: 77 (30–225) PS-CT: 415 (92–1781)	CS: 66 (8–425) CS-V: >1896 (49–1896) CS-CT: >2129 (46–2129)	Proportion local disease free: CS: 20% CS-V: 74% CS-CT: 89% Proportion metastasis free: CS: 45% CS-V: 84% CS-CT: 87% PS: 44% PS-CT: 80%	Edema & induration at vaccine site	[106]	3b

Abbreviations: cIFNβ: canine interferon-β, CS: complete surgery, CS-CT: complete surgery and combined therapy, CS-V: complete surgery and vaccination, hGM-CSF: human granulocyte–macrophage colony-stimulating factor, hIL: human interleukin, PNRCT: prospective non-randomized controlled clinical trial, PS: partial surgery, PS-CT: partial surgery and combined therapy, S: surgery group, S-CT: surgery and combined therapy.

**Table 14:** Summary of checkpoint inhibitor studies evaluating effectivity in oral malignant melanoma.

Overall Evidence Grade: B										
Immunotherapeutic Agent Evaluated	Primary Treatment	Adjunctive Therapy	Study Type	Number of Dogs	WHO Stage of Melanoma	Response Rate	Median Survival Time (Range) in Days	Adverse Events	Reference	LOE
Chimeric rat-dog anti-PD-L1 monoclonal antibody (c4G12)	NE	RT and/or surgery and/or CT	PNRCT (Using a HCG)	Total: 44 SG: 29 HCG: 15	SG: IV: 29 (100%) HCG: IV: 15 (100%)	Dogs with measurable disease (13/19) OR: 1/13 (8%) CR: 1/13 (8%) PD: 10/13 (77%) NE: 2/13 (15%)	SG: 143 (91–194) HCG: 54 (25-NA)	Anorexia: 1 Vomiting: 4 Diarrhea: 3 Thrombocytopaenia:2 Hypoalbuminaemia:1 Elevated ALT: 8 Elevated AST: 3 Elevated ALP: 1 Elevated lipase: 3 Elevated CPK: 1 Conjunctivitis: 1 Pneumonitis: 1	[108]	3b
Chimeric rat-dog (ch-4F12-E6) Or caninized (ca-4F12-E6) anti-PD-1 monoclonal antibodies	NE	Surgery and/or RT and/or CT and/or DNA vaccine	PNRCT (using a HCG)	Total: 44 SG: 21 HCG: 23	SG: III: 4 IV: 17 HCG: IV: 23	Stage IV dogs with measurable disease (15/17) OR: 4/15 (26.7%) PR: 4/15 (26.7%) PD: 10/15 (66.7%) SD: 1/15 (6.6%)	SG (IV): 166 (56–307) HCG (IV): 55 (27–143)	Fatigue: Grd 1: 3 Grd 2: 1 Anorexia: Grd 1: 3 Fever: Grd 1: 4 Grd 2: 1 GI toxicity: Grd 1: 12 Tachypnoea: Grd 1: 2 Grd 2: 1 Tremor Grd 1: 1 Death: 1 (pneumonitis)	[110]	3b
Chimeric rat-dog anti-PD-L1 monoclonal antibody (c4G12)	NE	Yes Surgery and/or RT and/or CT	PCS	Total OMM: 7	II: 1 (14%) II: 2 (29%) IV: 4 (57%)	II & III & IV: OR: 1/7 (14%) PR: 1/7 (14%) PD: 6/7 (86%)	NE	Diarrhea: Grd 1: 1	[109]	3b
			Stage IV OMM dogs then compared to a HCG =RCCS	SG: 4/7 HCG: 15	SG: IV: 4 (100%) HCG: IV: 15 (100%)	NE	SG: 94 (89–220) HCG: 54 (7–111)	As above	[109]	3b
Chimeric mouse-dog anti-PDPN monoclonal antibody (P38Bf)	Surgery	RT & CT: 1	PCS	3	I: 1 III: 1 IV: 1	SD: 1 PD: 1 PD: 1	NE	Increase in C-RP: 3 GI toxicity: Grd 2: 1	[111]	4c

Abbreviations: ALT: alanine aminotransferase, AST: aspartate aminotransferase, CPK: creatine phosphokinase, CR: complete response, C-RP: C-reactive protein, CT: chemotherapy, GI: gastrointestinal, Grd: grade, HCG: historical control group, NE: not evaluated, OR: overall response, PCS: prospective case series, PD: progressive disease, PNRCT: prospective non-randomized controlled clinical trial, PD-1: programmed cell death protein 1, PD-L1: programmed cell death ligand 1, PDPN: podoplanin, RCCS: retrospective controlled clinical study, PR: partial response, RT: radiation therapy, and SG: study group.

**Table 15:** Summary of bacterial inoculation studies evaluating effectivity in oral malignant melanoma.

Overall Evidence Grade: B										
Immunotherapeutic Agent Evaluated	Primary Treatment	Adjunctive Therapy	Study Type	Number of Dogs	WHO Stage of Melanoma	Median Survival Time (Range) in Days	Outcome (Other)	Adverse Events	Reference	LOE
Systemic *Corynebacterium* *parvum*	Surgery	No	PNRCT	89 SG: 42 CG: 47	SG: I: 17 (41%) II: 19 (45%) III: 6 (14%) CG: I: 21 (45%) II: 20 (42%) III: 6 (13%)	SG: I-III: 370 I: 360 II & III: 288 CG: I-III: 228 I: 559 II & III: 121	Death due to OMM: SG: 57% CG: 75%	Nausea, vomiting or diarrhea within 12 hr. of injection. Injection site inflammation: 6	[36]	2b
				SG: 42	SG:	SG:	SG: 57%	Inj. site inflammation: 6		
					I: 17 (41%)	I–III: 370				
					II: 19 (45%)	I: 360				
					III: 6 (14%)	II and III: 288				
				CG: 47	CG:	CG:	CG: 75%			
					I: 21 (45%)	I–III: 228				
					II: 20 (42%)	I: 559				
					III: 6 (13%)	II and III: 121				
Local *Corynebacterium parvum*	Surgery	No	PCS	8	NE	NE	All dogs died from metastasis and/or reoccurrence mostly within 6 months of injections	NE	[112]	4c

Abbreviations: CG: control group, Inj.: injection, NE: not evaluated, PC: prospective cohort, PCS: prospective case series, PNRCT: prospective non-randomized controlled clinical trial, and SG: study group.

**Table 16:** Summary of stimulatory cytokine studies evaluating effectivity in oral malignant melanoma.

Overall Evidence Grade: B											
Immunotherapeutic Agent Evaluated	Primary Treatment	Adjunctive Therapy	Study Type	Number of Dogs	WHO Stage of Melanoma	Response Rate	Median DFI (Range) in Days	Median Survival Time (Range) in Days	Adverse Events	Reference	LOE
L-MTP-PE alone or with rcGM-CSF	Surgery	No	PRDBCT	Total: 98 SG: 25 CG: 25	SG: I: 11 (44%) II: 8 (32%) III: 6 (24%) CG: I: 9 (36%) II: 11 (44%) III: 5 (20%)	NE	SG: I-III: 346 II & III: 152 I: NR II: 152 III: 85 CG: I-III: 174 II & III: 156 I: 396 II: 150 III: 147	SG: I-III: 504 II & III: 258 I: NR II: 254 III: 338 CG: I-III: 271 II & III: 243 I: 414 II: 293 III: 157	Elevation in body temp. (1–2 °C) lasting 1–4 h after treatment	[113]	1b
				SG: 24 CG: 24	SG: I: 13 (54%) II: 7 (29%) III: 4 (17%) CG: I: 12 (50%) II: 8 (33%) III: 4 (17%)	NE	SG: I-III: 212 II & III: 90 I: 489 II: 118 III: 35 CG: I-III: 290 II & III: 112 I: 532 II: 117 III: 92	SG: I-III: 498 II & III: 286 I: 573 II: 261 III: NR CG: I-III: 501 II & III: 306 I: NR II: 228 III: 275	Thrombocytopenia: 4 Anterior uveitis: 2 Lethargy & mild diarrhea: 2 Gastritis: 1 Polyuria & polydipsia: 1	[113]	1b
Interferon-alpha	Surgery	CT (carboplatin)	RCCS	20 SG: 17 CG: 3	II: 7 (35%) III: 12 (60%) IV: 1 (5%)	NE	NE	SG: 894 CG: 86	Mild- moderate myelosuppression (carboplatin)	[114]	2b
rhTNF and rhIL-2	NE	Surgery and/or RT and/or immunotherapy and/or HT	PCS	13	II: 7 (54%) III: 3 (23%) IV: 3 (23%)	OR 5/13 (38%)	NE	NE	Vomiting Diarrhea Fever Weakness 1 patient died (rhTNF)	[115]	4c

Abbreviations: CG: control group, CT: chemotherapy, HT: hyperthermia, L-MTP-PE: liposome encapsulated muramyl tripeptide-phosphatidylethanolamine, NE: not evaluated, NR: not reached, OR: overall response, PCS: prospective case series, PRDBCT: prospective, randomized, double-blinded controlled trial, RCCS: retrospective controlled clinical study, rcGM-CSF: recombinant canine granulocyte macrophage colony-stimulating factor, rhIL-2: recombinant human interleukin 2, rhTNF: recombinant human tumor necrosis factor, RT: radiotherapy, and SG: study group.

**Table 17:** Summary of nanotechnology studies evaluating effectivity in oral malignant melanoma.

Overall Evidence Grade: D									
Immunotherapeutic Agent Evaluated	Primary Treatment	Adjunctive Therapy	Study Type	Number of Dogs	WHO Stage of Melanoma	Survival Time in Days	Adverse Events	Reference	LOE
mNPH alone OR Plant-based VLP alone OR In combination	NE	No	Case report	1	NE	780		[117]	4d
		RT	Case report	2	NE	1 dog: 150 (Unrelated cause-tumor free) 1 dog: NR (Tumor free at 600 days)	NE	[117]	4d
		RT	Case report	1	NE	300 (Unrelated cause -tumor free)	None	[117]	4d
mNPH alone or with VLP	NE	No	Case report	1	NE	1350	None	[116]	4d
		HRT	Case report	1	NE	Alive & in remission at 540	None	[116]	4d

Abbreviations: HRT: hypo-fractionated radiation therapy, mNPH: magnetic iron oxide nanoparticle hyperthermia, NE: not evaluated, NR: not reached, RT: radiation therapy, and VLP: virus-like nanoparticle.

## References

[1] Nishiya A.T., Massoco C.O., Felizzola C.R., Perlmann E., Batschinski K., Tedardi M.V., Garcia J.S., Mendonça P.P., Teixeira T.F., Zaidan Dagli M.L. (2016). Comparative Aspects of Canine Melanoma. Vet. Sci..

[2] Wingo K. (2018). Histopathologic Diagnoses from Biopsies of the Oral Cavity in 403 Dogs and 73 Cats. J. Vet. Dent..

[3] Todoroff R.J., Brodey R.S. (1979). Oral and pharyngeal neoplasia in the dog: A retrospective survey of 361 cases. J. Am. Vet. Med. Assoc..

[4] Gorlin R.J., Barron C.N., Chaudhry A.P., Clark J.J. (1959). The oral and pharyngeal pathology of domestic animals: A study of 487 cases. Am. J. Vet. Res..

[5] Brodey R. (1960). A clinical and pathological study of 130 neoplasms of the mouth and pharynx in the dog. Am. J. Vet. Res..

[6] Weiss E., Frese K. (1974). Tumours of the skin. Bull. World Health Organ..

[7] López F., Rodrigo J.P., Cardesa A., Triantafyllou A., Devaney K.O., Mendenhall W.M., Hai-gentz M., Strojan P., Pellitteri P.K., Bradford C.R. (2016). Update on primary head and neck mucosal melanoma. Head Neck.

[8] Mort R.L., Jackson I.J., Patton E.E. (2015). The melanocyte lineage in development and disease. Development.

[9] Spangler W.L., Kass P.H. (2006). The histologic and epidemiologic bases for prognostic considerations in canine melanocytic neoplasia. Vet. Pathol..

[10] Ramos-Vara J.A., Beissenherz M.E., Miller M.A., Johnson G.C., Pace L.W., Fard A., Kottler S.J. (2000). Retrospective study of 338 canine oral melanomas with clinical, histologic, and immunohistochemical review of 129 cases. Vet. Pathol..

[11] Dorn C.R., Priester W.A. (1976). Epidemiologic analysis of oral and pharyngeal cancer in dogs, cats, horses, and cattle. J. Am. Vet. Med. Assoc..

[12] Vos J.H., van der Gaag I. (1987). Canine and feline oral-pharyngeal tumours. Zent. Vet. A.

[13] Svendenius L., Warfvinge G. (2010). Oral Pathology in Swedish Dogs: A Retrospective Study of 280 Biopsies. J. Vet. Dent..

[14] Bostock D.E. (1979). Prognosis after surgical excision of canine melanomas. Vet. Pathol..

[15] Bolon B., Calderwood Mays M.B., Hall B.J. (1990). Characteristics of canine melanomas and comparison of histology and DNA ploidy to their biologic behavior. Vet. Pathol..

[16] Harvey H.J., MacEwen E.G., Braun D., Patnaik A.K., Withrow S.J., Jongeward S. (1981). Prognostic criteria for dogs with oral melanoma. J. Am. Vet. Med. Assoc..

[17] McGill L.D., Blue J., Powers B. (2002). Report of the ad hoc committee on oncology to the ACVP membership and interested pathology community. American College of Veterinary Pathologists. Vet. Pathol..

[18] Esplin D.G. (2008). Survival of dogs following surgical excision of histologically well-differentiated melanocytic neoplasms of the mucous membranes of the lips and oral cavity. Vet. Pathol..

[19] Bergman P.J. (2007). Canine oral melanoma. Clin. Tech. Small Anim. Pract..

[20] Smedley R.C., Spangler W.L., Esplin D.G., Kitchell B.E., Bergman P.J., Ho H.Y., Bergin I.L., Kiupel M. (2011). Prognostic markers for canine melanocytic neoplasms: A comparative review of the literature and goals for future investigation. Vet. Pathol..

[21] Webster J.D., Dennis M.M., Dervisis N., Heller J., Bacon N.J., Bergman P.J., Bienzle D., Cassali G., Castagnaro M., Cullen J. (2011). Recommended guidelines for the conduct and evaluation of prognostic studies in veterinary oncology. Vet. Pathol..

[22] Tellado M.N., Maglietti F.H., Michinski S.D., Marshall G.R., Signori E. (2020). Electrochemotherapy in treatment of canine oral malignant melanoma and factors influencing treatment outcome. Radiol. Oncol..

[23] Elwood C., Devauchelle P., Elliott J., Freiche V., German A.J., Gualtieri M., Hall E., den Hertog E., Neiger R., Peeters D. (2010). Emesis in dogs: A review. J. Small Anim. Pract..

[24] Schwarz P.D., Withrow S.J., Curtis C.R., Powers B.E., Straw R.C. (1991). b. Partial maxillary resection as a treatment for oral cancer in 61 dogs. J. Am. Anim. Hosp. Assoc..

[25] Schwarz P.D., Withrow S.J., Curtis C.R., Powers B.E., Straw R.C. (1991). a. Mandibular resection as a treatment for oral cancer in 81 dogs. J. Am. Anim. Hosp. Assoc..

[26] Tuohy J.L., Selmic L.E., Worley D.R., Ehrhart N.P., Withrow S.J. (2014). Outcome following curative-intent surgery for oral melanoma in dogs: 70 cases (1998–2011). J. Am. Vet. Med. Assoc..

[27] Withrow S.J., Holmberg D.L. (1983). Mandibulectomy in the treatment of oral cancer. J. Am. Anim. Hosp. Assoc..

[28] Bradley R.L., MacEwen E.G., Loar A.S. (1984). Mandibular resection for removal of oral tumors in 30 dogs and 6 cats. J. Am. Vet. Med. Assoc..

[29] Beck E.R., Withrow S.J., McChesney A.E., Richardson R.C., Henderson R.A., Norris A.M., Caywood D.D., Klausner J.S., Harvey H.J., Holmberg D.L. (1986). Canine tongue tumors: A retrospective review of 57 cases. J. Am. Anim. Hosp. Assoc..

[30] Salisbury S.K., Lantz G.C. (1988). Long-term results of partial mandibulectomy for treatment of oral tumours in 30 dogs. J. Am. Anim. Hosp. Assoc..

[31] Kosovsky J.K., Matthiesen D.T., Marretta S.M., Patnaik A.K. (1991). Results of partial mandibulectomy for the treatment of oral tumors in 142 dogs. Vet. Surg..

[32] Syrcle J.A., Bonczynski J.J., Monette S., Bergman P.J. (2008). Retrospective evaluation of lingual tumors in 42 dogs: 1999–2005. J. Am. Anim. Hosp. Assoc..

[33] Culp W.T., Ehrhart N., Withrow S.J., Rebhun R.B., Boston S., Buracco P., Reiter A.M., Schallberger S.P., Aldridge C.F., Kent M.S. (2013). Results of surgical excision and evaluation of factors associated with survival time in dogs with lingual neoplasia: 97 cases (1995–2008). J. Am. Vet. Med. Assoc..

[34] Sarowitz B.N., Davis G.J., Kim S. (2017). Outcome and prognostic factors following curative-intent surgery for oral tumours in dogs: 234 cases (2004 to 2014). J. Small Anim. Pract..

[35] Brockley L.K., Cooper M.A., Bennett P.F. (2013). Malignant melanoma in 63 dogs (2001–2011): The effect of carboplatin chemotherapy on survival. N. Z. Vet. J..

[36] MacEwen E.G., Patnaik A.K., Harvey H.J., Hayes A.A., Matus R. (1986). Canine oral melanoma: Comparison of surgery versus surgery plus *Corynebacterium parvum*. Cancer Investig..

[37] Boston S.E., Lu X., Culp W.T., Montinaro V., Romanelli G., Dudley R.M., Liptak J.M., Mestrinho L.A., Buracco P. (2014). Efficacy of systemic adjuvant therapies administered to dogs after excision of oral malignant melanomas: 151 cases (2001–2012). J. Am. Vet. Med. Assoc..

[38] Hahn K., DeNicola D.B., Richardson R.C., Hahn E.A. (1994). Canine oral malignant melanoma: Prognostic utility of an alternative staging system. J. Small Anim. Pract..

[39] Withrow S.J., Vail D.M., Page R.L. (2012). Withrow & MacEwen’s Small Animal Clinical Oncology.

[40] Giannakakou P., Sackett D., Fojo T. (2000). Tubulin/microtubules: Still a promising target for new chemotherapeutic agents. J. Natl. Cancer Inst..

[41] Rassnick K.M., Ruslander D.M., Cotter S.M., Al-Sarraf R., Bruyette D.S., Gamblin R.M., Meleo K.A., Moore A.S. (2001). Use of carboplatin for treatment of dogs with malignant melanoma: 27 cases (1989–2000). J. Am. Vet. Med. Assoc..

[42] Kitchell B.E., Brown D.M., Luck E.E., Woods L.L., Orenberg E.K., Bloch D.A. (1994). Intralesional implant for treatment of primary oral malignant-melanoma in dogs. J. Am. Vet. Med. Assoc..

[43] Dank G., Rassnick K.M., Sokolovsky Y., Garrett L.D., Post G.S., Kitchell B.E., Sellon R.K., Kleiter M., Northrup N., Segev G. (2014). Use of adjuvant carboplatin for treatment of dogs with oral malignant melanoma following surgical excision. Vet. Comp. Oncol..

[44] Ogilvie G.K., Obradovich J.E., Elmslie R.E., Vail D.M., Moore A.S., Straw R.C., Dickinson K., Cooper M.F., Withrow S.J. (1991). Efficacy of mitoxantrone against various neoplasms in dogs. J. Am. Vet. Med. Assoc..

[45] Boria P.A., Murry D.J., Bennett P.F., Glickman N.W., Snyder P.W., Merkel B.L., Schlittler D.L., Mutsaers A.J., Thomas R.M., Knapp D.W. (2004). Evaluation of cisplatin combined with piroxicam for the treatment of oral malignant melanoma and oral squamous cell carcinoma in dogs. J. Am. Vet. Med. Assoc..

[46] Rutteman G.R., Erich S.A., Mol J.A., Spee B., Grinwis G.C., Fleckenstein L., London C.A., Efferth T. (2013). Safety and efficacy field study of artesunate for dogs with non-resectable tumours. Anticancer Res..

[47] Giuliano A., Dobson J. (2020). Prospective clinical trial of masitinib mesylate treatment for advanced stage III and IV canine malignant melanoma. J. Small Anim. Pract..

[48] Ji-Yun L., Sang-Yeon O., Chul P., Hun-Young Y., Soon-Wuk J., Hee-Myung P. (2003). Oral malignant melanoma in a Labrador retriever. J. Vet. Clin..

[49] Hajduch M., Kolár Z., Novotný R., Hanus J., Mihál V., Hlobílková A., Nosková V., Strnad M. (1997). Induction of apoptosis and regression of spontaneous dog melanoma following in vivo application of synthetic cyclin-dependent kinase inhibitor olomoucine. Anti-Cancer Drugs.

[50] Murphy S., Hayes A.M., Blackwood L., Maglennon G., Pattinson H., Sparkes A.H. (2005). Oral malignant melanoma—The effect of coarse fractionation radiotherapy alone or with adjuvant carboplatin therapy. Vet. Comp. Oncol..

[51] Cunha S.C.D., Corgozinho K.B., Silva F.B.F., da Silva K.V.G.C., Ferreira A.M.R. (2018). Radiation therapy for oral melanoma in dogs: A retrospective study. Cienc. Rural.

[52] Freeman K.P., Hahn K.A., Harris F.D., King G.K. (2003). Treatment of dogs with oral melanoma by hypofractionated radiation therapy and platinum-based chemotherapy (1987–1997). J. Vet. Intern. Med..

[53] Kawabe M., Mori T., Ito Y., Murakami M., Sakai H., Yanai T., Maruo K. (2015). Outcomes of dogs undergoing radiotherapy for treatment of oral malignant melanoma: 111 cases (2006–2012). J. Am. Vet. Med..

[54] Proulx D.R., Ruslander D.M., Dodge R.K., Hauck M.L., Williams L.E., Horn B., Price G.S., Thrall D.E. (2003). A retrospective analysis of 140 dogs with oral melanoma treated with external beam radiation. Vet. Radiol. Ultrasound.

[55] Tollett M.A., Duda L., Brown D.C., Krick E.L. (2016). Palliative radiation therapy for solid tumors in dogs: 103 cases (2007–2011). J. Am. Vet. Med. Assoc..

[56] LaDue T.A., Dodge R., Page R.L., Price G.S., Hauck M.L., Thrall D.E. (1999). Factors influencing survival after radiotherapy of nasal tumors in 130 dogs. Vet. Radiol. Ultrasound.

[57] Blackwood L., Dobson J.M. (1996). Radiotherapy of oral malignant melanomas in dogs. J. Am. Vet. Med. Assoc..

[58] Bateman K.E., Catton P.A., Pennock P.W., Kruth S.A. (1994). 0-7-21 radiation therapy for the treatment of canine oral melanoma. J. Vet. Intern. Med..

[59] Theon A.P., Rodriguez C., Madewell B.R. (1997). Analysis of prognostic factors and patterns of failure in dogs with malignant oral tumors treated with megavoltage irradiation. J. Am. Vet. Med. Assoc..

[60] Esmaeili N., Friebe M. (2019). Electrochemotherapy: A review of current status, alternative IGP approaches, and future perspectives. J. Healthc. Eng..

[61] Spugnini E.P., Dragonetti E., Vincenzi B., Onori N., Citro G., Baldi A. (2006). Pulse-mediated chemotherapy enhances local control and survival in a spontaneous canine model of primary mucosal melanoma. Melanoma Res..

[62] Maglietti F., Tellado M., Olaiz N., Michinski S., Marshall G. (2016). Combined local and systemic bleomycin administration in electrochemotherapy to reduce the number of treatment sessions. Radiol. Oncol..

[63] Kulbacka J., Paczuska J., Rembiałkowska N., Saczko J., Kiełbowicz Z., Kinda W., Liszka B., Kotulska M., Kos B., Miklavčič D. (2017). Electrochemotherapy combined with standard and Co2 laser surgeries in canine oral melanoma. Slov. Vet. Zb..

[64] Maglietti F.H., Michinski S.D., Ricotti I., Maure P., Mir L.M., Olaiz N., Marshall G. (2014). Amelanotic melanoma of the root of the tongue in a canine patient treated by electrochemotherapy. J. Anal. Oncol..

[65] Dewhirst M.W., Sim D.A., Forsyth K., Grochowski K.J., Wilson S., Bicknell E. (1985). Local control and distant metastases in primary canine malignant melanomas treated with hyperthermia and/or radiotherapy. Int. J. Hyperth..

[66] Thompson J.M., Turrel J.M. (1987). Hyperthermia and Radiation in the Management of Canine Tumors. J. Small Anim. Pract..

[67] Theon A.P., Madewell B.R., Moore A.S., Stephens C., Krag D.N. (1991). Localized thermo-cisplatin therapy—a pilot-study in spontaneous canine and feline tumors. Int. J. Hyperth..

[68] Dressel S., Gosselin M.C., Capstick M.H., Carrasco E., Weyland M.S., Scheidegger S., Neufeld E., Kuster N., Bodis S., Rohrer Bley C. (2018). Novel hyperthermia applicator system allows adaptive treatment planning: Preliminary clinical results in tumour-bearing animals. Vet. Comp. Oncol..

[69] Lucroy M.D., Chen W.R., Ridgway T.D., Higbee R.G., Bartels K.E. (2002). Selective laser-induced hyperthermia for the treatment of spontaneous tumors in dogs. J. X-ray Sci. Technol..

[70] Saleem M., Kaur S., Kweon M.H., Adhami V.M., Afaq F., Mukhtar H. (2005). Lupeol, a fruit and vegetable based triterpene, induces apoptotic death of human pancreatic adenocarcinoma cells via inhibition of Ras signaling pathway. Carcinogenesis.

[71] Nitta M., Azuma K., Hata K., Takahashi S., Ogiwara K., Tsuka T., Imagawa T., Yokoe I., Osaki T., Minami S. (2013). Systemic and local injections of lupeol inhibit tumor growth in a melanoma-bearing mouse model. Biomed. Rep..

[72] Saleem M., Maddodi N., Abu Zaid M., Khan N., bin Hafeez B., Asim M., Suh Y., Yun J.M., Setaluri V., Mukhtar H. (2008). Lupeol inhibits growth of highly aggressive human metastatic melanoma cells in vitro and in vivo by inducing apoptosis. Clin. Cancer Res..

[73] Chaturvedi P.K., Bhui K., Shukla Y. (2008). Lupeol: Connotations for chemoprevention. Cancer Lett..

[74] Yokoe I., Azuma K., Hata K., Mukaiyama T., Goto T., Tsuka T., Imagawa T., Itoh N., Murahata Y., Osaki T. (2015). Clinical systemic lupeol administration for canine oral malignant melanoma. Mol. Clin. Oncol..

[75] Withers S.S., York D., Johnson E., Al-Nadaf S., Skorupski K.A., Rodriguez C.O., Burton J.H., Guerrero T., Sein K., Wittenburg L. (2018). In vitro and in vivo activity of liposome-encapsulated curcumin for naturally occurring canine cancers. Vet. Comp. Oncol..

[76] Bergman P.J. (2019). Cancer Immunotherapies. Vet. Clin. Small Anim..

[77] Bergman P.J. (2014). Immunotherapy in veterinary oncology. Vet. Clin. N. Am. Small Anim. Pract..

[78] Catchpole B., Gould S.M., Kellett-Gregory L.M., Dobson J.M. (2002). Immunosuppressive cytokines in the regional lymph node of a dog suffering from oral malignant melanoma. J. Small Anim. Pract..

[79] Almela R.M., Ansón A. (2019). A review of immunotherapeutic strategies in canine malignant melanoma. Vet. Sci..

[80] Regan D., Guth A., Coy J., Dow S. (2016). Cancer immunotherapy in veterinary medicine: Current options and new developments. Vet. J..

[81] Treggiari E., Grant J.P., North S.M. (2016). A retrospective review of outcome and survival following surgery and adjuvant xenogeneic DNA vaccination in 32 dogs with oral malignant melanoma. J. Vet. Med. Sci..

[82] McLean J.L., Lobetti R.G. (2015). Use of the melanoma vaccine in 38 dogs: The South African experience. J. S. Afr. Vet. Assoc..

[83] Grosenbaugh D.A., Leard A.D., Bergman P.J., Klein M.K., Meleo K., Susaneck S., Hess P.R., Jankowski M.K., Jones P.D., Leibman N.F. (2011). Safety and efficacy of a xenogeneic DNA vaccine encoding for human tyrosinase as adjunctive treatment for oral malignant melanoma in dogs following surgical excision of the primary tumor. Am. J. Vet. Res..

[84] Ottnod J.M., Smedley R.C., Walshaw R., Hauptman J.G., Kiupel M., Obradovich J.E. (2013). A retrospective analysis of the efficacy of Oncept vaccine for the adjunct treatment of canine oral malignant melanoma. Vet. Comp. Oncol..

[85] Turek M., LaDue T., Looper J., Nagata K., Shiomitsu K., Keyerleber M., Buchholz J., Gieger T., Hetzel S. (2020). Multimodality treatment including ONCEPT for canine oral melanoma: A retrospective analysis of 131 dogs. Vet. Radiol. Ultrasound.

[86] Verganti S., Berlato D., Blackwood L., Amores-Fuster I.., Polton G.A., Elders R., Doyles R., Taylor A., Murphy S. (2017). Use of Oncept melanoma vaccine in 69 canine oral malignant melanomas in the UK. J. Small Anim. Pract..

[87] Gyorffy S., Rodriguez-Lecompte J.C., Woods J.P., Foley R., Kruth S., Liaw P.C.Y., Gauldie J. (2005). Bone marrow-derived dendritic cell vaccination of dogs with naturally occurring melanoma by using human gp100 antigen. J. Vet. Intern. Med..

[88] Alexander A.N., Huelsmeyer M.K., Mitzey A., Dubielzig R.R., Kurzman I.D., MacEwen E.G., Vail D.M. (2006). Development of an allogeneic whole-cell tumor vaccine expressing xenogeneic gp100 and its implementation in a phase II clinical trial in canine patients with malignant melanoma. Cancer Immunol. Immunother..

[89] Zuleger C.L., Kang C., Ranheim E.A., Kurzman I.D., Macklin M.D., Newton M.A., Wolchock J.D., Vail D.M., Eriksson E., Albertini M.R. (2017). Pilot study of safety and feasibility of DNA microseeding for treatment of spontaneous canine melanoma. Vet. Med. Sci..

[90] Nemec A., Milevoj N., Tratar U.L., Sersa G., Cemazar M., Tozon N. (2020). Electroporation-based treatments in small animal veterinary oral and maxillofacial oncology. Front. Vet. Sci..

[91] Riccardo F., Lussich S., Maniscalco L., Lorda Mayayo S., La Rosa G., Arigoni M., De Maria R., Gattino F., Lanzardo S., Lardone E. (2014). CSPG4-specific immunity and survival prolongation in dogs with oral malignant melanoma immunized with human CSPG4 DNA. Clin. Cancer Res..

[92] Piras L.A., Riccardo F., Iussich S., Maniscalco L., Gattino F., Martano M., Morello E., Lorda Mayayo S., Rolih V., Garavaglia F. (2017). Prolongation of survival of dogs with oral malignant melanoma treated by en bloc surgical resection and adjuvant CSPG4-antigen electrovaccination. Vet. Comp. Oncol..

[93] Giacobino D., Camerino M., Riccardo F., Cavallo F., Tarone L., Martano M., Dentini A., Iussich S., Lardone E., Franci P. (2021). Difference in outcome between curative intent vs marginal excision as a first treatment in dogs with oral malignant melanoma and the impact of adjuvant CSPG4-DNA electrovaccination: A retrospective study on 155 cases. Vet. Comp. Oncol..

[94] Thamm D.H., Kurzman I.D., Mac Ewen E.G., Feinmehl R., Towell T.L., Longhofer S.L., Johnson C.M., Geoly F.J., Stinchcomb D.T. (2003). Intralesional lipid-complexed cytokine/superantigen immunogene therapy for spontaneous canine tumors. Cancer Immunol. Immunother..

[95] Finocchiaro L.M., Fiszman G.L., Karara A.L., Glikin G.C. (2008). Suicide gene and cytokines combined nonviral gene therapy for spontaneous canine melanoma. Cancer Gene Ther..

[96] Quintin-Colonna F., Devauchelle P., Fradelizi D., Mourot B., Faure T., Koueilsky P., Roth C., Mehtali M. (1996). Gene therapy of spontaneous canine melanoma and feline fibrosarcoma by intratumoral administration of histoincompatible cells expressing human interleukin-2. Gene Ther..

[97] Westberg S., Sadeghi A., Svensson E., Segall T., Dimopoulou M., Korsgren O., Hemminki A., Loskog A.S.I., Totterman T.H., von Euler H. (2013). Treatment efficacy and immune stimulation by AdCD40L gene therapy of spontaneous canine malignant melanoma. J. Immunother..

[98] Von Euler H., Sadeghi A., Carlsson B., Rivera P., Loskog A., Segall T., Korsgren O., Totterman T.H. (2008). Efficient adenovector CD40 ligand immunotherapy of canine malignant melanoma. J. Immunother..

[99] Dow S.W., Elmslie R.E., Willson A.P., Roche L., Gorman C., Potter T.A. (1998). In Vivo tumor transfection with superantigen plus cytokine genes induces tumor regression and prolongs survival in dogs with malignant melanoma. J. Clin. Investig..

[100] Bianco S.R., Sun J., Fosmire S.P., Hance K., Padilla M.L., Ritt M.G., Getzy D.M., Duke R.C., Withrow S.J., Lana S. (2003). Enhancing antimelanoma immune responses through apoptosis. Cancer Gene Ther..

[101] Cicchelero L., Denies S., Vanderperren K., Stock E., Van Brantegem L., de Rooster H., Sanders N.N. (2017). Immunological, anti-angiogenic and clinical effects of intratumoral interleukin 12 electrogene therapy combined with metronomic cyclophosphamide in dogs with spontaneous cancer: A pilot study. Cancer Lett..

[102] Cutrera J., King G., Jones P., Kicenuik K., Gumpel E., Xia X., Li S. (2015). Safety and efficacy of tumor-targeted interleukin 12 gene therapy in treated and non-treated, metastatic lesions. Curr. Gene Ther..

[103] Reed S.D., Fulmer A., Buckholz J., Zhang B., Cutrera J., Shiomitsu K., Li S. (2010). Bleomycin/interleukin-12 electrochemogene therapy for treating naturally occurring spontaneous neoplasms in dogs. Cancer Gene Ther..

[104] Milevoj N., Tratar U.L., Nemec A., Brozic A., Znidar K., Sersa G., Cemazar M., Tozon N. (2019). A combination of electrochemotherapy, gene electrotransfer of plasmid encoding canine IL-12 and cytoreductive surgery in the treatment of canine oral malignant melanoma. Res. Vet. Sci..

[105] Finocchiaro L.M., Fondello C., Gil-Cardeza M.L., Rossi U.A., Villaverde M.S., Riveros M.D., Glikin G.C. (2015). Cytokine-enhanced vaccine and interferon-β plus suicide gene therapy as surgery adjuvant treatments for spontaneous canine melanoma. Hum. Gene Ther..

[106] Finocchiaro L.M.E., Agnetti L., Fondello C., Glikin G.C. (2019). Combination of cytokine-enhanced vaccine and chemo-gene therapy as surgery adjuvant treatments for spontaneous canine melanoma. Gene Ther..

[107] Esfahani K., Roudaia L., Buhlaiga N., Del Rincon S.V., Papneja N., Miller W.H. (2020). A review of cancer immunotherapy: From the past, to the present, to the future. Curr. Oncol..

[108] Maekawa N., Konnai S., Nishimura M., Kagawa Y., Takagi S., Hosoya K., Ohta H., Kim S., Okagawa T., Izumi Y. (2021). PD-L1 immunohistochemistry for canine cancers and clinical benefit of anti-PD-L1 antibody in dogs with pulmonary metastatic oral malignant melanoma. NPJ Precis. Oncol..

[109] Maekawa N., Konnai S., Takagi S., Kagawa Y., Okagawa T., Nishimori A., Ikebuchi R., Izumi Y., Deguchi T., Nakajima C. (2017). A canine chimeric monoclonal antibody targeting PD-L1 and its clinical efficacy in canine oral malignant melanoma or undifferentiated sarcoma. Sci. Rep..

[110] Igase M., Nemoto Y., Itamoto K., Tani K., Nakaichi M., Sakurai M., Sakai Y., Noguchi S., Kato M., Tsukui T. (2020). A pilot clinical study of the therapeutic antibody against canine PD-1 for advanced spontaneous cancers in dogs. Sci. Rep..

[111] Kamoto S., Shinada M., Kato D., Yoshimoto S., Ikeda N., Tsuboi M., Yoshitake R., Eto S., Hashimoto Y., Takahashi Y. (2020). Phase I/II clinical trial of the anti-podoplanin monoclonal antibody therapy in dogs with malignant melanoma. Cells.

[112] Misdorp W. (1987). Incomplete surgery, local immunostimulation, and recurrence of some tumour types in dogs and cats. Vet. Q..

[113] MacEwen E.G., Kurzman I.D., Vail D.M., Dubielzig R.R., Everlith K., Madewell B.R., Rodriguez Jr. C.O., Phillips B., Zwahlen C.H., Obradovich J. (1999). Adjuvant therapy for melanoma in dogs: Results of randomized clinical trials using surgery, liposome-encapsulated muramyl tripeptide, and granulocyte macrophage colony-stimulating factor. Clin. Cancer Res..

[114] Lavalle G.E., Caires C.E.T., Teixeira S.V., Cunha R.M.C., Carneiro R.A. (2021). Treatment of canine oral melanoma with adjuvant chemotherapy and immunotherapy. Acta Sci. Vet..

[115] Moore A.S., Theilen G.H., Newell A.D., Madewell B.R., Rudolf A.R. (1991). Preclinical study of sequential tumor necrosis factor and interleukin 2 in the treatment of spontaneous canine neoplasms. Cancer Res..

[116] Hoopes P.J., Moodie K.L., Petryk A.A., Petryk J.D., Sechrist S., Gladstone D.J., Steinmetz N.F., Veliz F.A., Bursey A.A., Wagner R.J. (2017). Hypo-fractionated radiation, magnetic nanoparticle hyperthermia and a viral immunotherapy treatment of spontaneous canine cancer. Proc. SPIE Int. Soc. Opt. Eng..

[117] Hoopes P.J., Wagner R.J., Duval K., Kang K., Gladstone D.J., Moodie K.L., Crary-Burney M., Ariaspulido H., Veliz F.A., Steinmetz N.F. (2018). Treatment of canine oral melanoma with nanotechnology-based immunotherapy and radiation. Mol. Pharm..

[118] Pernick N. Staging-Mucosal Melanoma. https://www.pathologyoutlines.com/topic/oralcavitystagingmucosalmelanoma.html.

[119] Edge S., Compton C.C. (2009). AJCC Cancer Staging Manual.

[120] Kamstock D.A., Ehrhart E.J., Getzy D.M., Bacon N.J., Rassnick K.M., Moroff S.D., Liu S.M., Straw R.C., McKnight C.A., Amorim R.L. (2011). Recommended guidelines for submission, trimming, margin evaluation, and reporting of tumor biopsy specimens in veterinary surgical pathology. Vet. Pathol..

[121] Scolyer R.A., Rawson R.V., Gershenwald J.E., Ferguson P.M., Prieto V.G. (2020). Melanoma pathology reporting and staging. Mod. Pathol..

[122] Liptak J.M. (2020). Histologic margins and the residual tumour classification scheme: Is it time to use a validated scheme in human oncology to standardise margin assessment in veterinary oncology?. Vet. Comp. Oncol..

